# Supplementary material for: A natural biological adhesive from snail mucus for wound repair
Source: Nat Commun. 2023 Jan 24;14:396. doi: 10.1038/s41467-023-35907-4 (PMC9873654; doi:10.1038/s41467-023-35907-4)
Supplement: Supplementary file 1 — Supplementary Information [file 41467_2023_35907_MOESM1_ESM.pdf]

Supplementary Information

A Natural Biological Adhesive from Snail Mucus for Wound Repair

Tuo Deng, Dongxiu Gao, Xuemei Song, Zhipeng Zhou, Lixiao Zhou, Maixian Tao, Zexiu Jiang, Lian Yang, Lan Luo, Ankun Zhou, Lin Hu, Hongbo Qin & Mingyi Wu*

*Corresponding author: Mingyi Wu

Email: wumingyi@mail.kib.ac.cn

This PDF file includes:

Supplementary Methods

Supplementary Figures 1–17

Supplementary Tables 1–12

Supplementary References 1–3

Supplementary Methods

1. Characteristic staining analysis of d-SMG

1.1 Staining analysis of polymers and calcium salt in d-SMG

Fresh snail mucus was evenly smeared on glass slide. After drying naturally, the slide was immersed in 5% formaldehyde solution for 5 h. Then, the slide was dried and immersed in 1,9-dimethylmethylene blue staining solution, Coomassie brilliant blue staining solution or Alizarin red staining solution for 5 min. Then, slide was rinsed and dried. After that, the photos of stained SMGs were captured via Carl Zeiss Microscopy GmbH (Axio Scan Z1, Germany).

1.2 Arnow staining: identification of L-Dopa

To test the presence of L-3,4-dihydroxyphenylalanine (L-Dopa) in the snail mucus, Arnow staining was performed. The d-SMG samples (10.0 mg) were accurately weighed and hydrolyzed in 2 mL HCl (6 M) for 22 h at 110 ℃. The pH was adjusted to 6 ~ 8 with NaOH (10 M). Then, the solution was diluted to 10.0 mL with deionized water, the diluent was centrifuged at 4000×g for 15 min, and the supernatant was stained with nitrite - molybdic acid solution (Arnow reaction)[1]. L-Dopa solution (10 μg/mL) was used as a positive control.

2. Compositional Analysis of d-SMG

2.1 Protein molecular weight analysis by SDS-PAGE

2.0 mg d-SMG was accurately weighed and dissolved in 2.0 mL PBS, and the supernatant was collected after 15000 × g centrifugation. Then solution was denatured by boiling at 100 °C for 10 min. After mixing with loading buffer containing bromophenol blue, the marker and d -SMG proteins were separated by a 10% polyacrylamide gel. The protein in the gel was stained by Coomassie brilliant blue and photos were captured.

2.2 Quantification of total protein in d-SMG

The protein content of d-SMG was measured by using the Bicinchoninic Acid (BCA) Protein Assay Kit (Solarbio, Beijing China). Bovine Serum Albumin (BSA) was used as the standard substance and the equation of linear regression was established according to the kit instruction. 25.0 mg d-SMG was accurately weighed and dissolved in 25 mL PBS. After centrifugation (15000 × g, 15 min), the absorbance of the protein in supernatant was detected, and protein content was calculated from the standard curve

2.3 Quantification of amino acids in d-SMG

The d-SMG (10 ~ 20 mg) were accurately weighed and hydrolyzed in 10 mL HCl (6 M) at 110 ℃ for 22 ~ 24 h. The solution was diluted to 25.00 mL, and 1.00 mL solution was evaporated in a water bath at 60 ℃.

The samples were redissolved in sample diluent and the amino acid content was determined by an amino acid analyzer (A300, Membra Pure GmbH, Germany). The test results are exported by the Chromatography Data Handling System.

2.4 FTIR spectra analysis of d-SMG and s-GAG

FTIR spectra (4000–400 cm^{-1}) were recorded using an FTIR spectrometer (Nicolet iS10, Thermo Scientific, USA) by the KBr compression method, and spectral data were recorded as absorbance units. PeakFit v4.12 software was applied to analyze the secondary structure, where the raw spectra (1600–1700 cm^{-1}) of amide I were deconvoluted, peak-split, and curve-fitted by Gaussian functions.

2.5 Quantification of s-GAG in d-SMG

The 1,9-dimethylmethylene blue assay and carbazole method efficient methods in measuring the sulfated glycosaminoglycan content[2,3]. The s-GAG was obtained as described in **Extraction and Purification of the s-GAG** in text. The concentration of s-GAG was measured as previously reported[2,3] by using ultraviolet (UV) spectrophotometry and pure snail GAG and glucuronic acid as control.

2.6 Quantification of elements in d-SMG

The d-SMG was pulverized to powder for 15 min in an agate mortar. Then the content of C, H, O, N, S, and P in d-SMG was tested by Elementar (Vario EL cube, Germany). Approximately 2 mg d-SMG was added into 2.0 mL nitric acid (67%) and 0.5 mL H_2O_2 (30%), after heating at 180 °C for 40 minthe mixture was cooled to room temperature. Then, the internal standard element Cs (0.1 mL, 1.0 μg/mL) was added, and the solution was diluted to 100.0 mL with 1% nitric acid. The contents of inorganic elements (Na, K, Mg, Ca, Fe, etc.) were determined by ICP–OES730 (Agilent, USA) and ICP–MS7800 (Agilent, USA).

2.7 Quantification of allantoin in d-SMG

The allantoin content of d-SMG was measured by high-performance liquid chromatography (HPLC, Agilent 1100, USA) with a hydrophilic chromatographic column (HILIC, Phenomenex Luna®, USA), which had the capability of partially retaining polar compounds. Standard curve was established with 0.040, 0.060, 0.080, 0.120, and 0.160 mg/mL allantoin. 25.0 mg d-SMG was dissolved in 25 mL deionized water and heated in a water bath at 60 ℃ for 15 min. Then the solution was stirred in a vortex device for approximately 2 min, and cooled to room temperature. After centrifugation (15000 × g, 15 min), and the supernatant was subjected to HPLC analysis, and the allantoin content was calculated from the standard curve.

2.8 NMR analysis of s-GAG in d-SMG

The NMR analysis was performed at 298 K in D_2O with a Bruker Avance spectrometer of 800 MHz equipped with a $^{13}C/^{1}H$ dual probe in FT mode. All samples were dissolved in D_2O (99.9%) at 10~20 mg/ml, and spectra were recorded with HDO suppression by presaturation.

2.9 Analysis of proteins in d-SMG by mass spectrometry

Briefly, d-SMG proteins were extracted and digested, and the obtained peptides were analyzed and detected by liquid chromatography tandem mass spectrometry (LC-MS/MS) (LC: Easy-nLC 1200, Thermo Fisher Scientific, USA; MS: Q Exactive™ Hybrid Quadrupole-Orbitrap™ Mass Spectrometer, Thermo Fisher Scientific, USA). In addition, proteins from two d-SMGs were separated by SDS-PAGE. The proteins from SDS-PAGE bands were also analyzed by LC-MS after enzymatic hydrolysis. The MS data were analyzed based on two protein databases (https://www.uniprot.org/uniprotkb?query=Stylommatophora and https://www.uniprot.org/uniprotkb?query=Gastropoda%20mucin) by using Maxquant (1.6.2.10). The complete data are available in Source Data and/or ProteomeXchange with identifier PXD036781 (Reviewer account details: Username: reviewer_pxd036781@ebi.ac.uk, Password: R83ttMLM).

2.10 Roman spectroscopy analysis of the interaction of d-SMG and tissue

Two pieces of mouse skin tissue were adhered with a layer of d-SMG. Then the adhered tissues were cut into 10 μm slices by Frozen histopathological section system (CryoStar NX50, ThermoFisher Scientific, USA). Raman spectra were recorded with an inVia Raman microscope (Renishaw, UK) using a 532 nm laser. The visual field of the adhesion area between tissue and d-SMG was selected and scanned.

Supplementary Figures

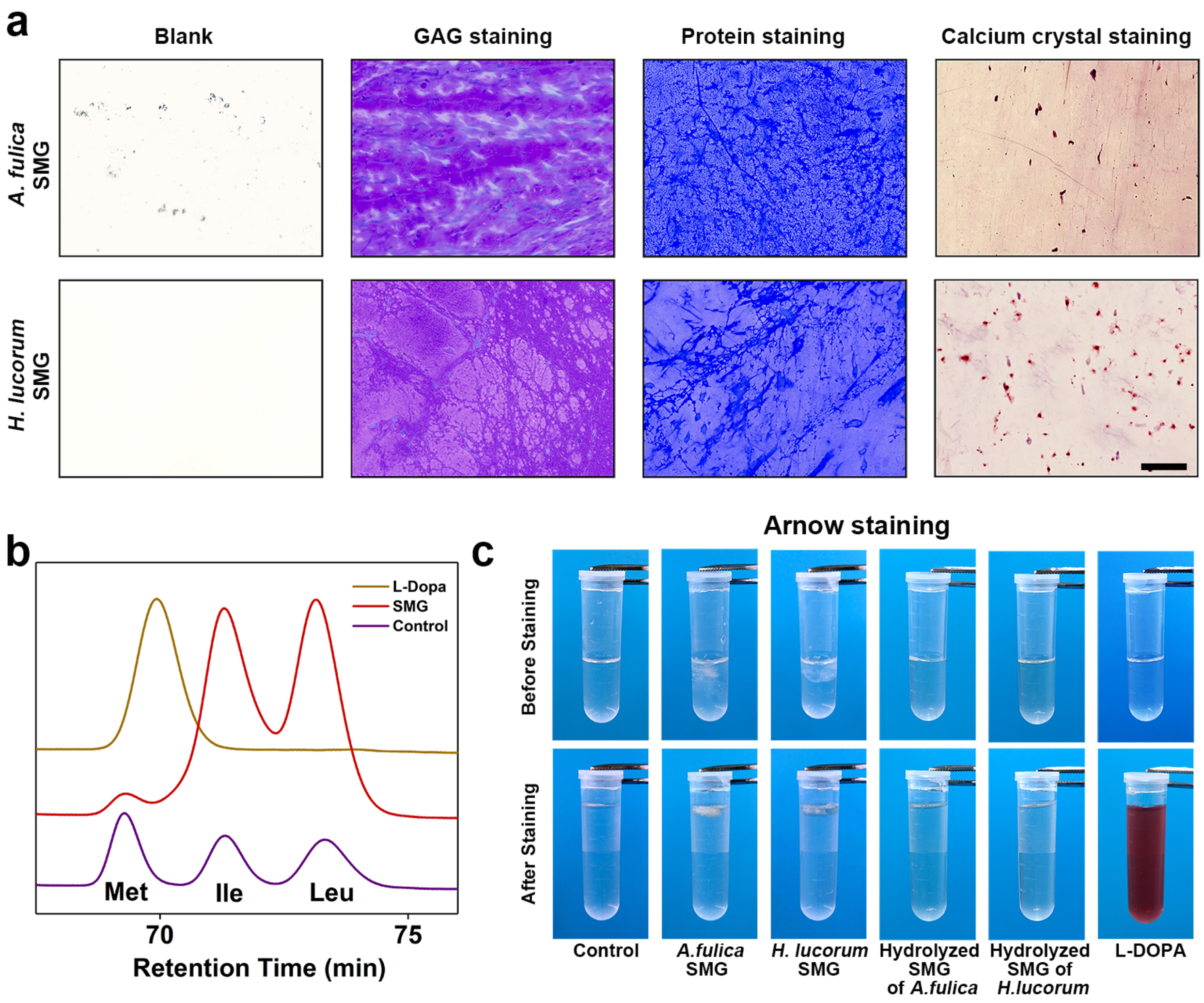

Supplementary Fig. 1. Qualitative identification of the main components of SMGs. a Staining of protein, GAG, and calcium salt in SMG (n = 1 biologically independent sample in each group). **b** Representative graph of amino acid analysis. **c** Arnow staining of SMG and hydrolyzed SMG. Scale bars, 100 μm (images in **a**). Source data are provided as a Source data file.

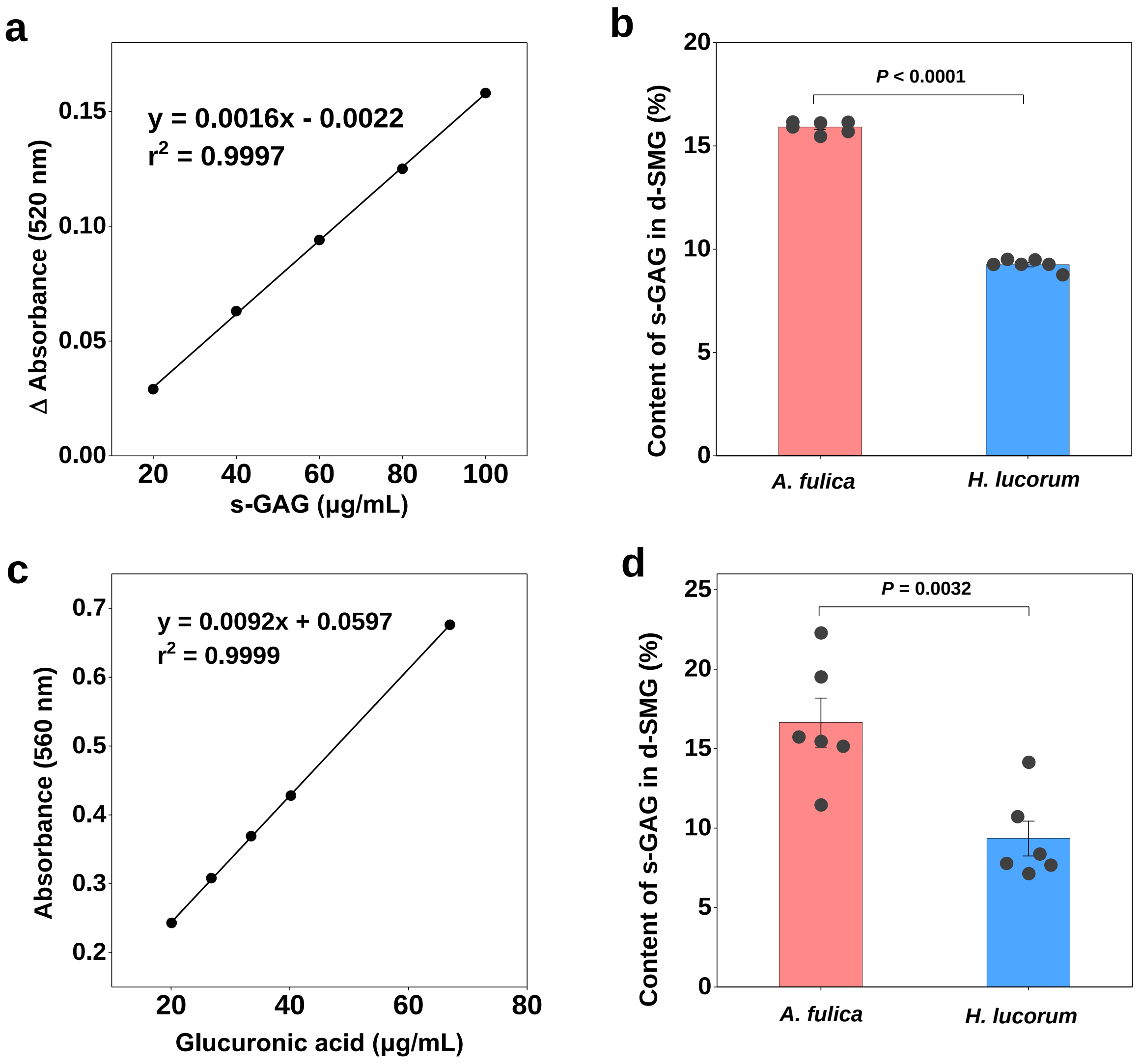

Supplementary Fig. 2. s-GAG content in two d-SMGs. a-b ΔA_{520nm} - s-GAG concentration standard curve (**a**) and the content of s-GAG in two d-SMGs (**b**) determined by 1,9-dimethylmethylene blue assay (*n* = 5 biologically independent samples in each group). **c-d** A_{560nm} - glucuronic acid concentration standard curve (**c**) and the content of s-GAG in two d-SMGs (**d**) determined by carbazole method (*n* = 5 biologically independent samples in each group). For **b** and **d**, two-tailed t test was used. Data are presented as mean values +/- SEM. Source data are provided as a Source data file.

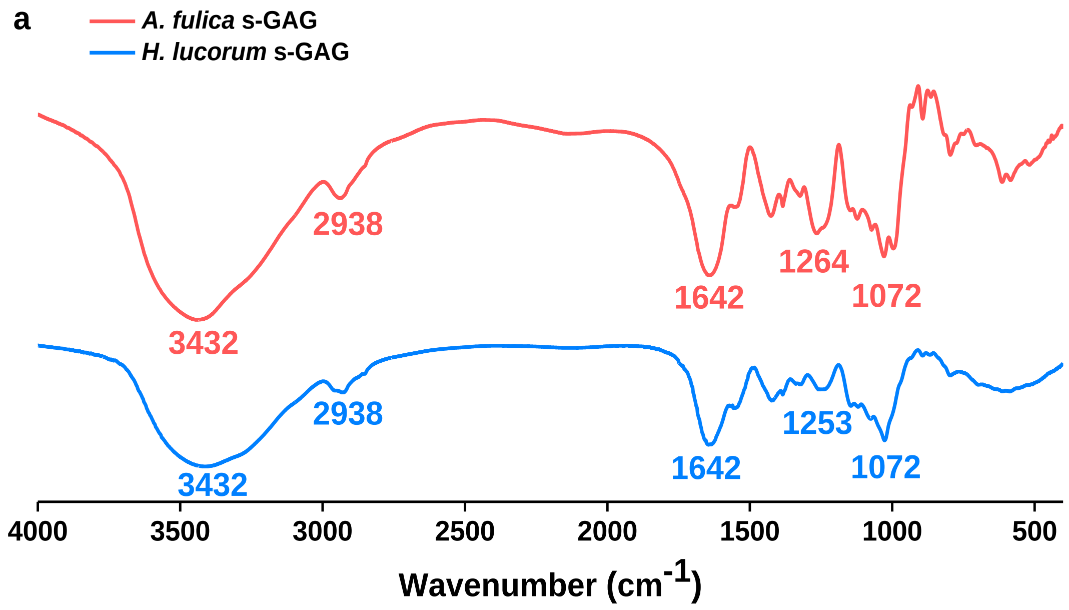
a
A. fulica s-GAG
H. lucorum s-GAG
2938
3432
1642
1264
1072
2938
3432
1642
1253
1072
4000
3500
3000
2500
2000
1500
1000
500
Wavenumber (cm-1)

b

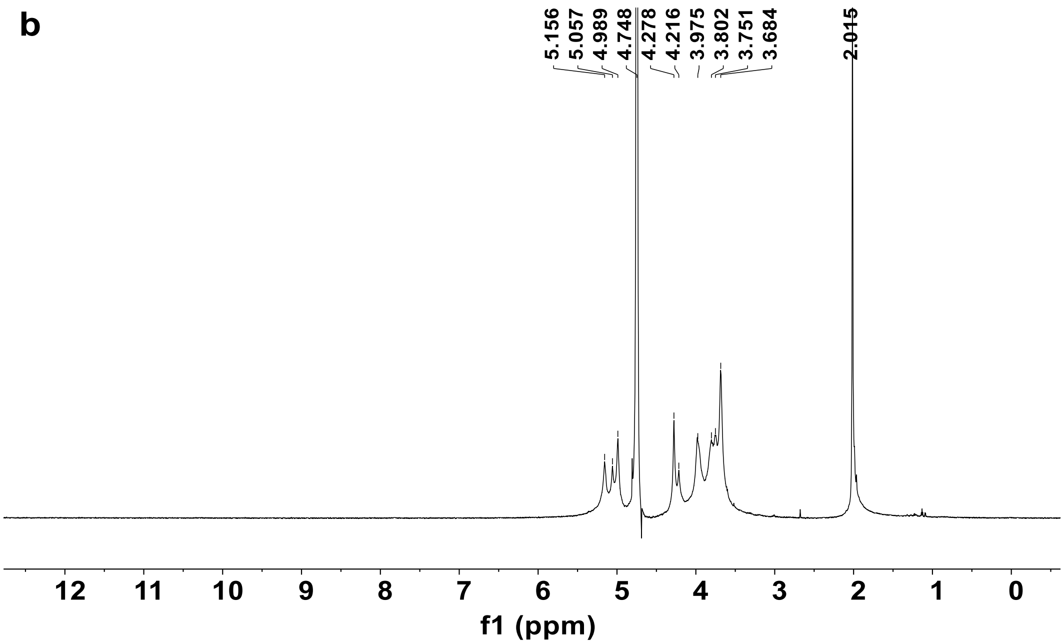

5.156
5.057
4.989
4.748
4.278
4.216
3.975
3.802
3.751
3.684
2.015
12 11 10 9 8 7 6 5 4 3 2 1 0
f1 (ppm)

c

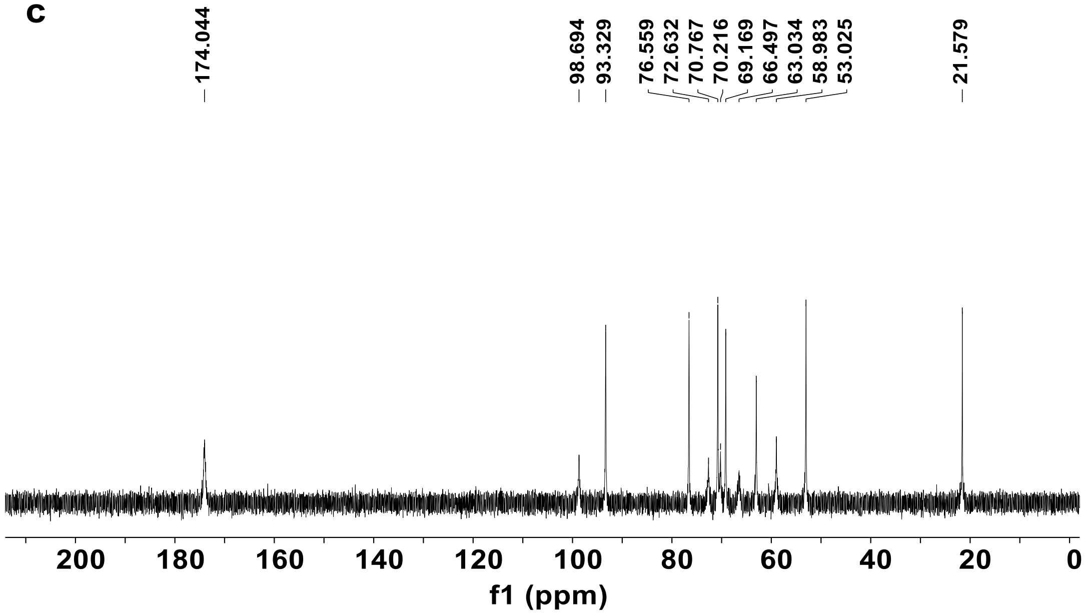

174.044
98.694
93.329
76.559
72.632
70.767
70.216
69.169
66.497
63.034
58.983
53.025
21.579
200 180 160 140 120 100 80 60 40 20 0
f1 (ppm)

d

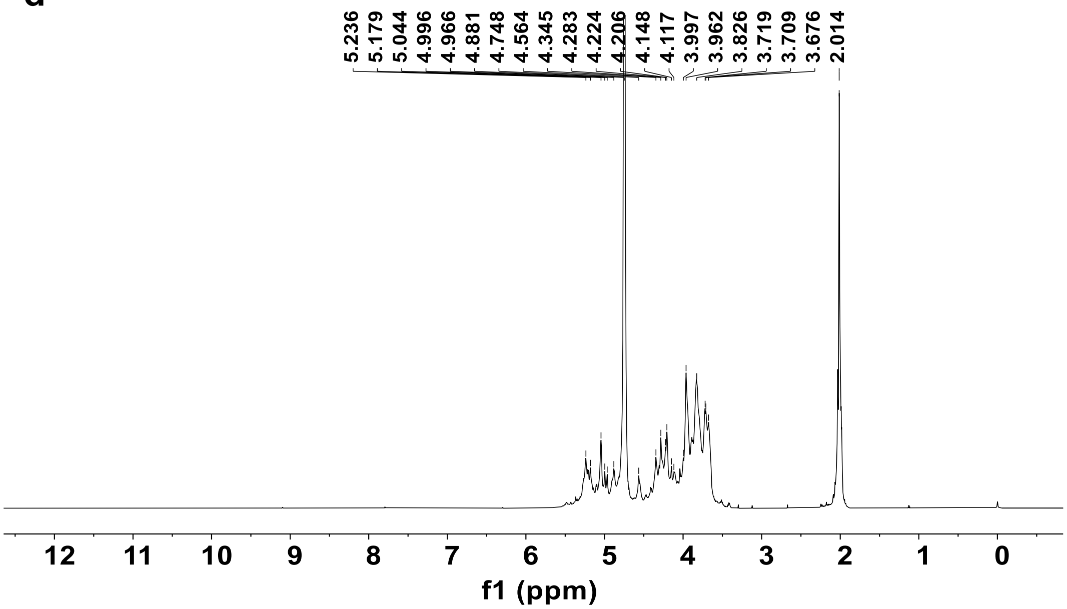

e

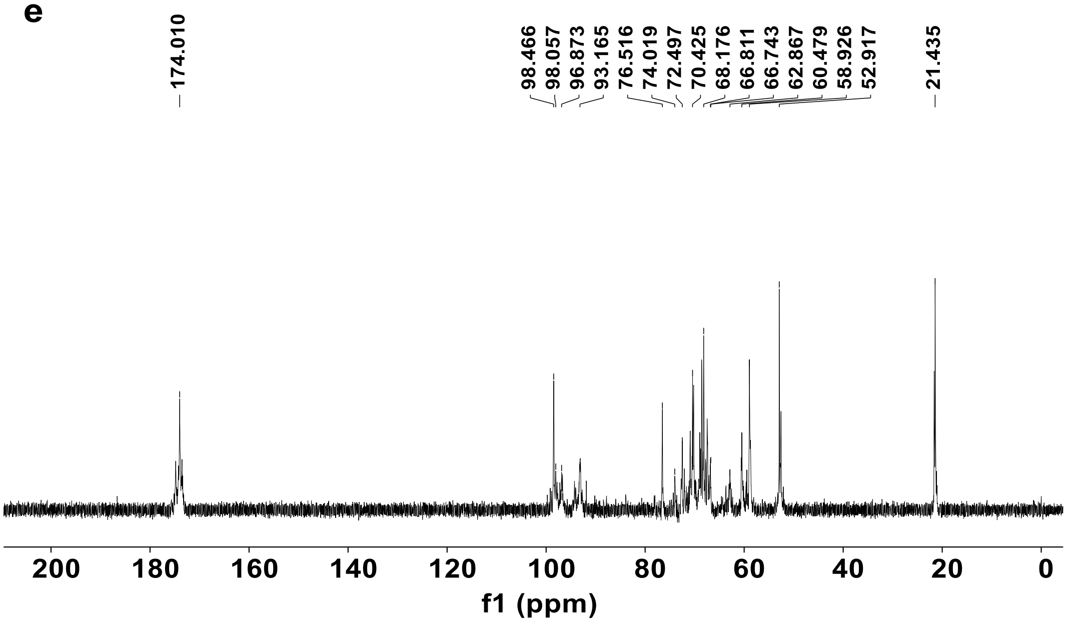

Supplementary Fig. 3. Structure characteristics of s-GAG from two d-SMGs. a FT-IR spectra of s-GAG. **b** ^{1}H NMR of *A. fulica* s-GAG. **c** ^{13}C NMR of *A. fulica* s-GAG. **d** ^{1}H NMR of *H. lucorum* s-GAG. **e** ^{13}C NMR of *H. lucorum* s-GAG.

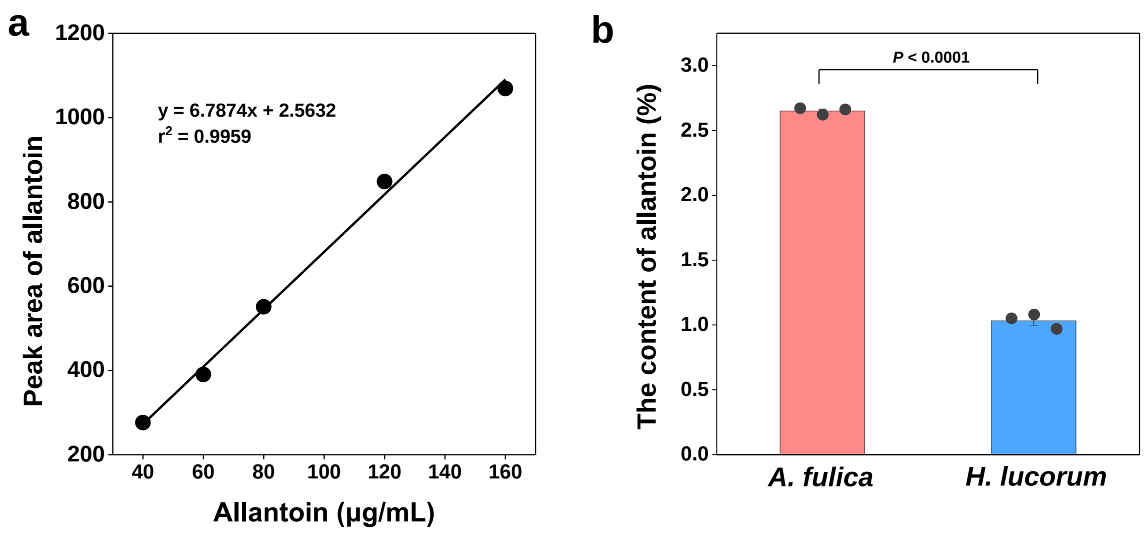

Supplementary Fig. 4. Allantoin content in two d-SMGs. a Peak area-allantoin concentration standard curve determined by HPLC. **b** The content of allantoin in two d-SMGs (n = 3 biologically independent samples in each group). For **b**, two-tailed t test was used. Data are presented as mean values +/- SEM. Source data are provided as a Source data file.

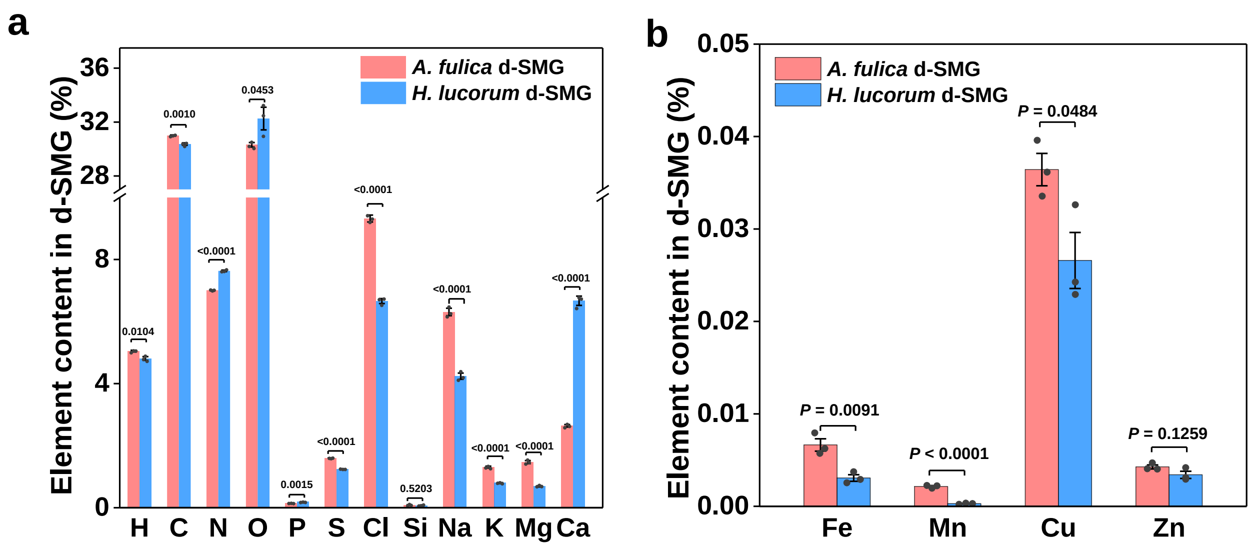

Supplementary Fig. 5. Element content in two d-SMGs. **a** The content of main organic elements and metallic elements in d-SMG (*n* = 3 biologically independent samples in each group). **b** Trace amounts of metallic elements in d-SMG (*n* = 3 biologically independent samples in each group). For **a** and **b**, two-tailed t test was used. Data are presented as mean values +/- SEM. Source data are provided as a Source data file.

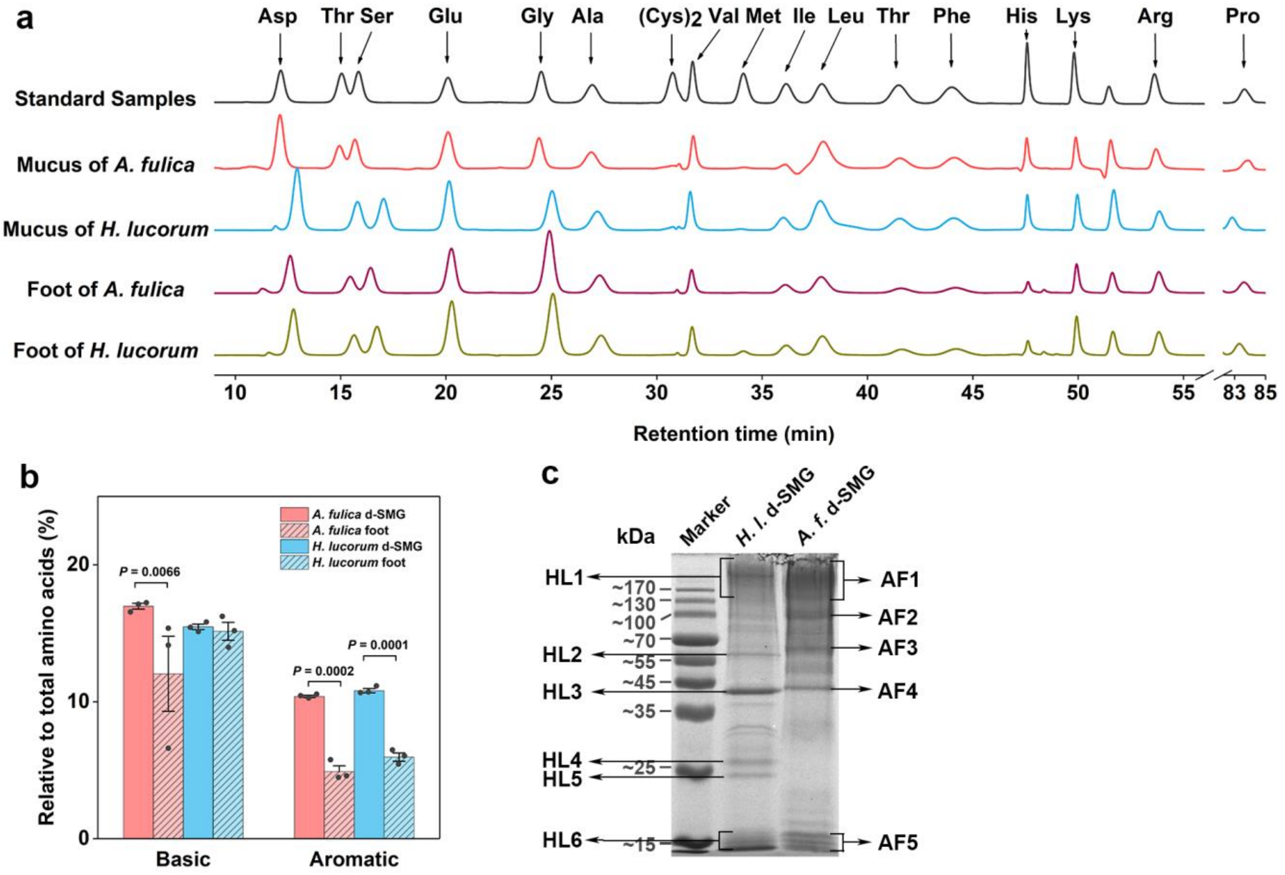

Supplementary Fig. 6. The protein and amino acid compositions in d-SMGs. a Amino acid compositions of snail mucus and foot detected by acid hydrolysis and ion chromatography assay. **b** The basic and aromatic amino acids contents in d-SMG (*n* = 3 biologically independent samples in each group). **c** SDS-PAGE image of protein from d-SMG detected by Coomassie bright blue staining (*n* = 3 biologically independent samples in each group). For **b**, two-tailed t test was used. Data are presented as mean values +/- SEM. Source data are provided as a Source data file.

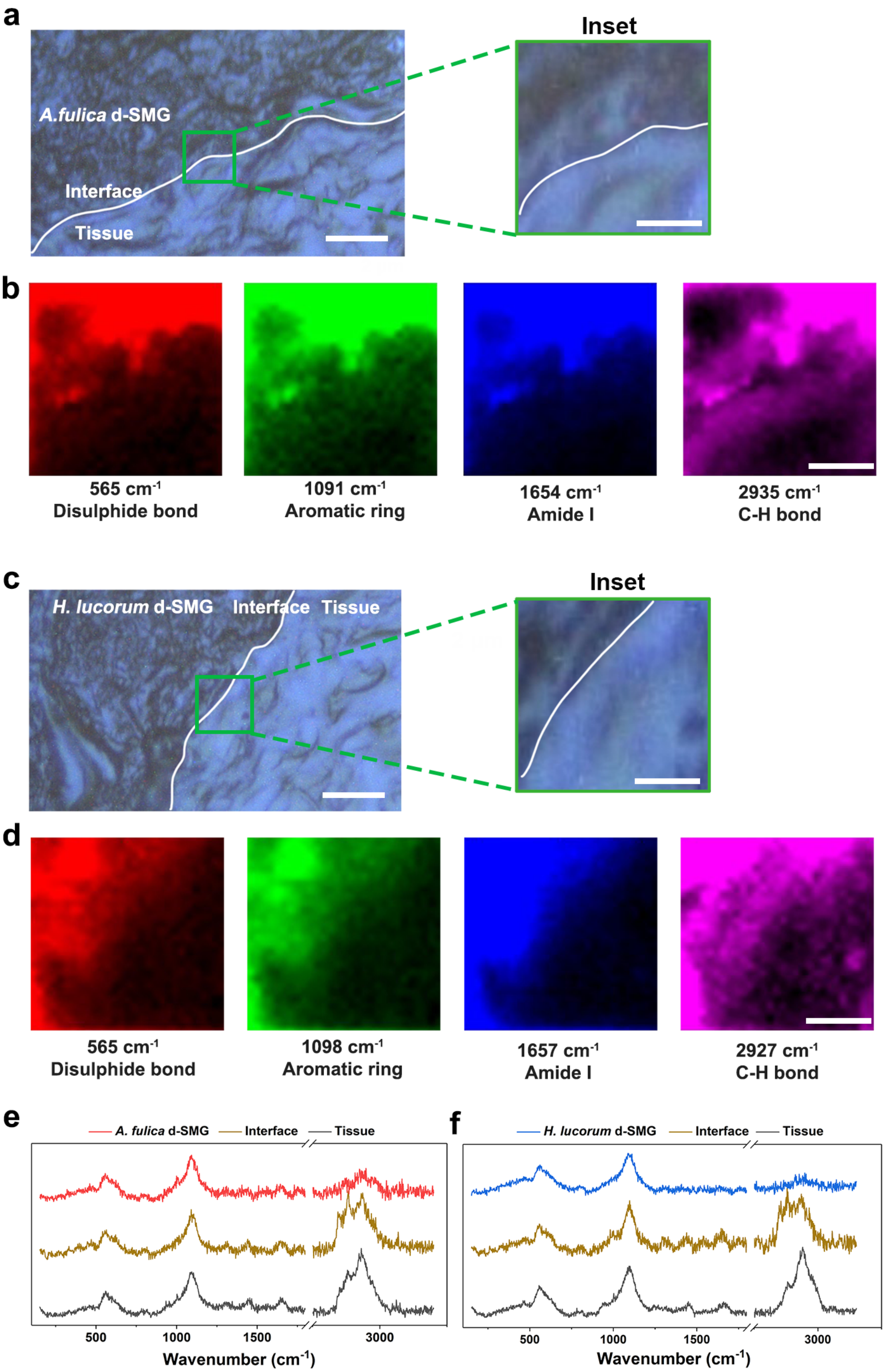

a
Inset
A.fulica d-SMG
Interface
Tissue
b
565 cm-1
Disulphide bond
1091 cm-1
Aromatic ring
1654 cm-1
Amide I
2935 cm-1
C-H bond
c
Inset
H. lucorum d-SMG
Interface
Tissue
d
565 cm-1
Disulphide bond
1098 cm-1
Aromatic ring
1657 cm-1
Amide I
2927 cm-1
C-H bond
e
A. fulica d-SMG
Interface
Tissue
500
1000
1500
3000
Wavenumber (cm-1)
f
H. lucorum d-SMG
Interface
Tissue
500
1000
1500
3000
Wavenumber (cm-1)

Supplementary Fig. 7. Raman spectroscopy analysis of the interaction between d-SMG and tissue. a and **c** Raman spectrum scanning area of d-SMG, interface, and tissue. **b** and **d** Raman mapping images of the d-SMG, tissue, and their interface. **e** and **f** Raman spectra of the d-SMG, tissue, and their interface. Scale bars, 2 μm (images in **a, c**), 0.5 μm (inset in **a, c** and images in **b, d**). Source data are provided as a Source data file.

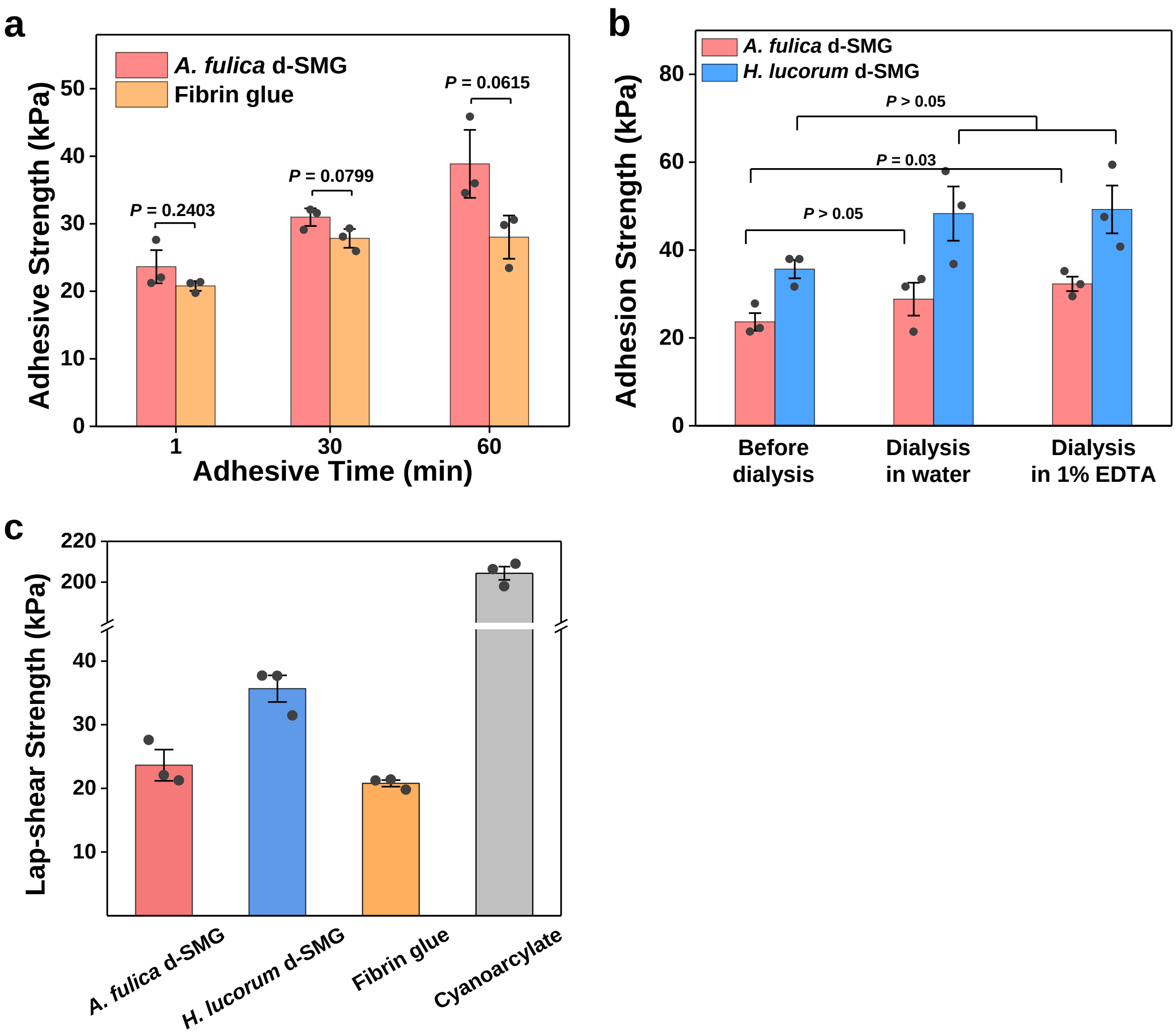

Supplementary Fig. 8. Adhesive properties of d-SMGs. Effects of adhesion time **(a)** and Ca^{2+} content **(b)** on the adhesion strength of d-SMG. **c** Adhesion strength of different commercial adhesives and d-SMG. For **a**–**c**, two-tailed t test was used, *n* = 3 biologically independent samples in each group. Data are presented as mean values +/- SEM. Source data are provided as a Source data file.

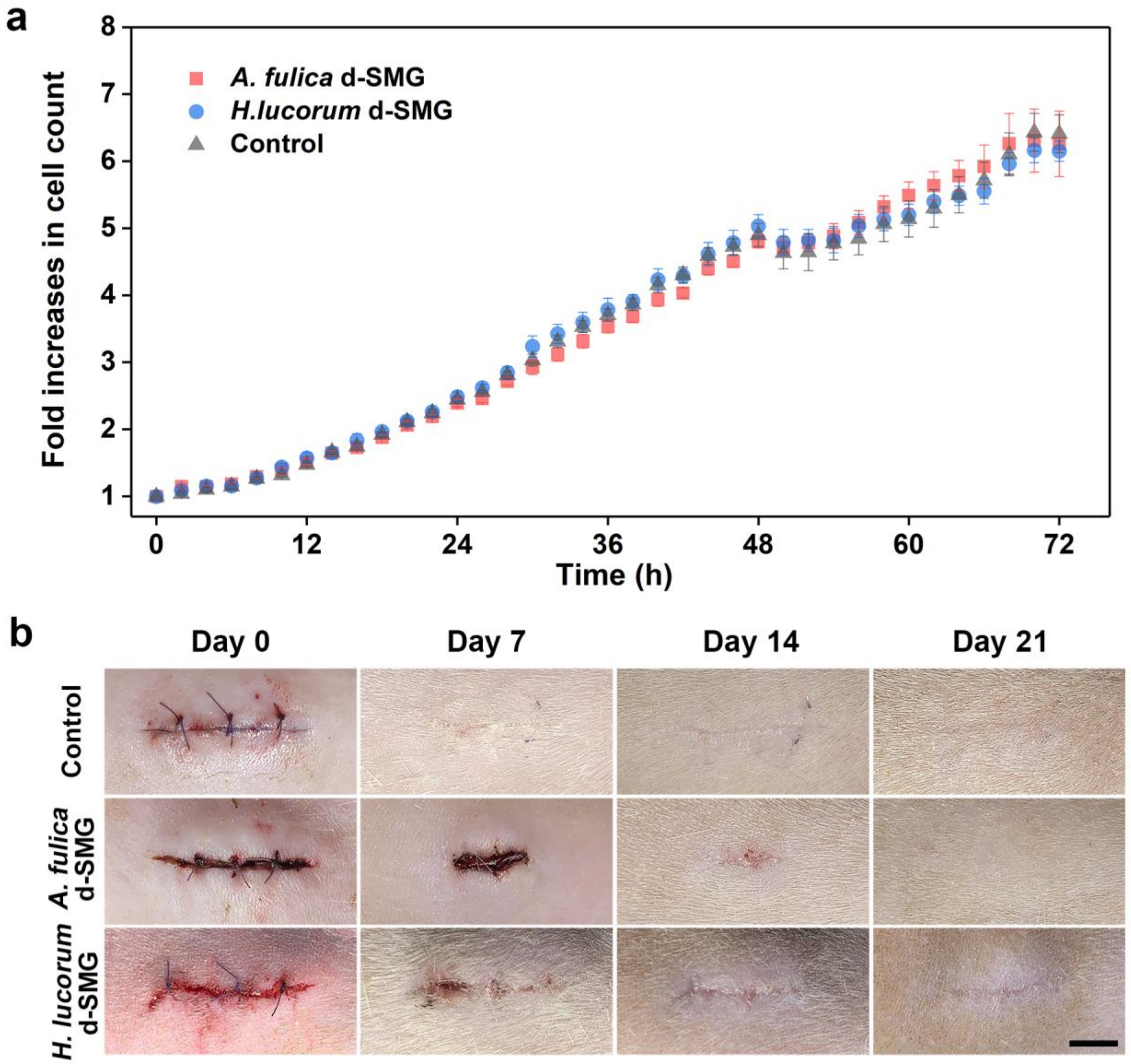

Supplementary Fig. 9. The cytocompatibility of d-SMG. a Effect of d-SMGs on the cell growth of L929. **b** Representative images of d-SMG subcutaneous implantation in rat skin. For **a**, data were expressed as mean ± SEM, *n* = 5. Scale bar, 5 mm (images in **b**). Source data are provided as a Source data file.

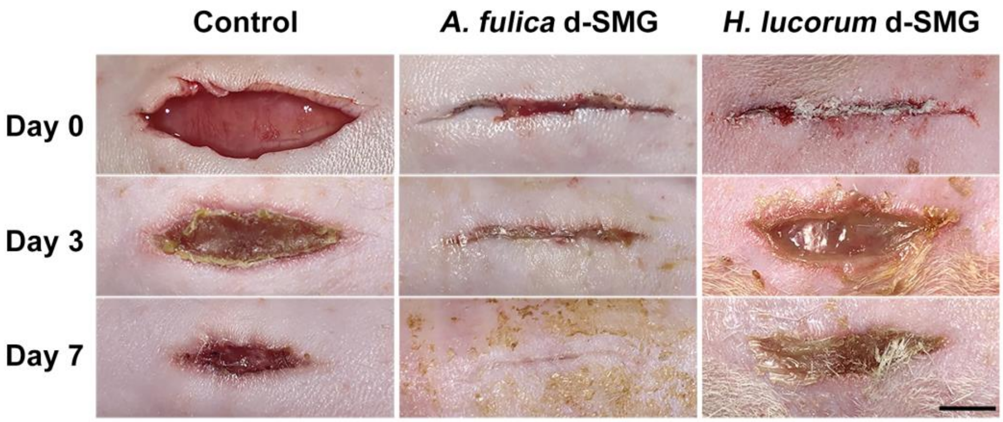

Supplementary Fig. 10. The *in vivo* tissue adhesion effect of d-SMGs. Representative images of rat skin incision treated with the *A. fulica* d-SMG and the *H. lucorum* d-SMG on day 0, 3, and 7. Scale bar, 5 mm.

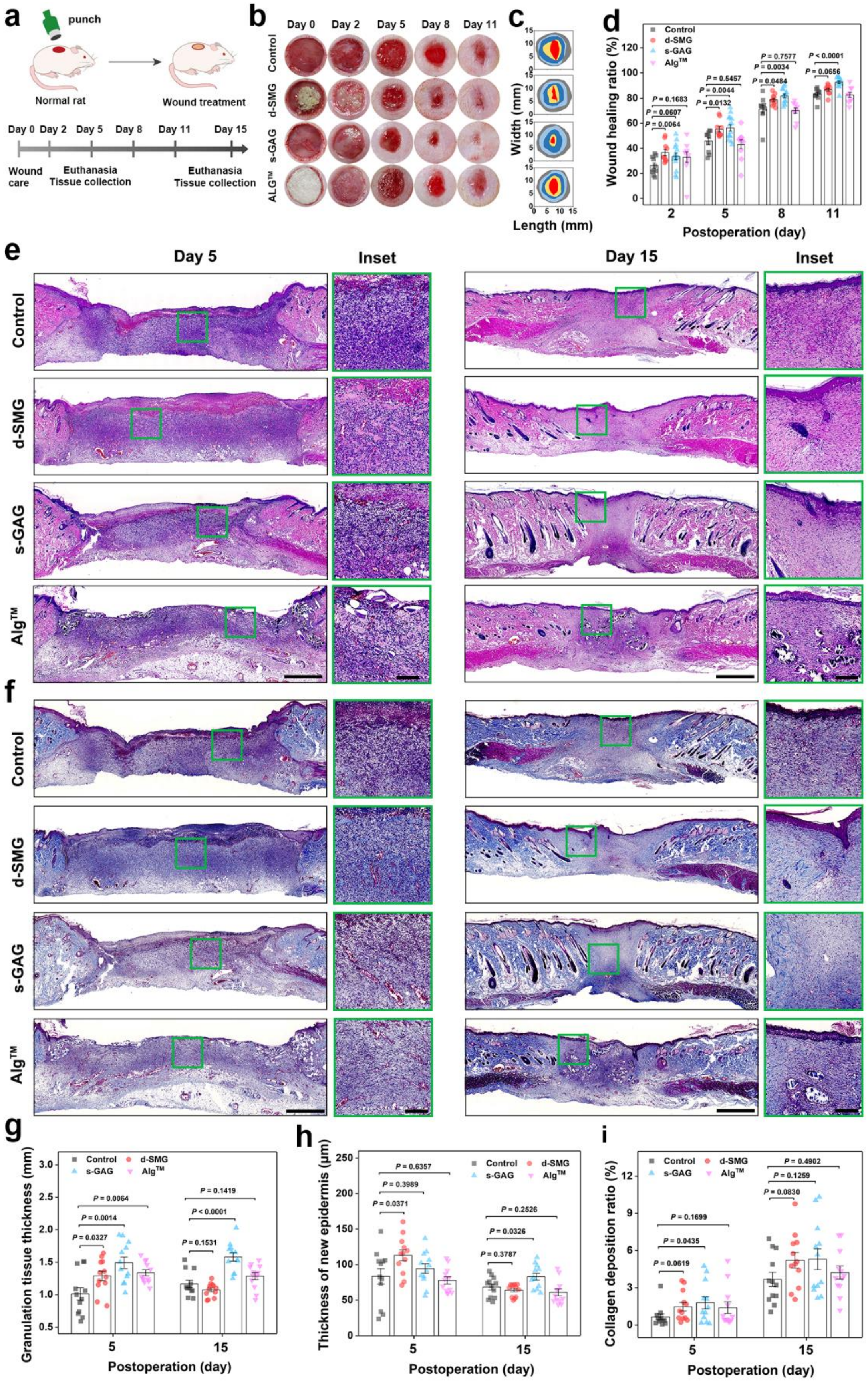

a
punch
Normal rat
Wound treatment
Day 0 Day 2 Day 5 Day 8 Day 11 Day 15
Wound care
Euthanasia Tissue collection
Euthanasia Tissue collection
b
Day 0 Day 2 Day 5 Day 8 Day 11
Control
d-SMG
s-GAG
ALG™
c
Width (mm)
Length (mm)
d
Control
d-SMG
s-GAG
Alg™
Wound healing ratio (%)
Postoperation (day)
e
Day 5
Inset
Day 15
Inset
f
g
Granulation tissue thickness (mm)
Postoperation (day)
h
Thickness of new epidermis (μm)
Postoperation (day)
i
Collagen deposition ratio (%)
Postoperation (day)

Supplementary Fig. 11. Wound healing efficacies of *A. fulica* d-SMG in normal rats. a Schematic illustration of the wound model in normal SD rats. **b** Representative images of the wound during the healing process. **c** Schematic diagram of the dynamic wound healing process. **d** The wound healing ratio after treatments (*n* = 5 rats created over 2 independent wounds in Control, d-SMG, and Alg^{TM} group, *n* = 7 rats created over 2 independent wounds in s-GAG group). **e–f** H&E-staining (**e**) and Masson staining (**f**) images of the wound tissue on day 5 and 15. **g-i** Quantification of the granulation tissue thickness (**g**), neo epidermis thickness (**h**) and collagen deposition ratio (**i**) in wound tissue on day 5 and 15 (*n* = 12 biologically independent samples in each group). For **d**, **g–i**, two-tailed t test was used. Data are presented as mean values +/- SEM. Scale bars, 1 mm (images in **e, f**), 200 μm (inset in **e, f**). Source data are provided as a Source data file.

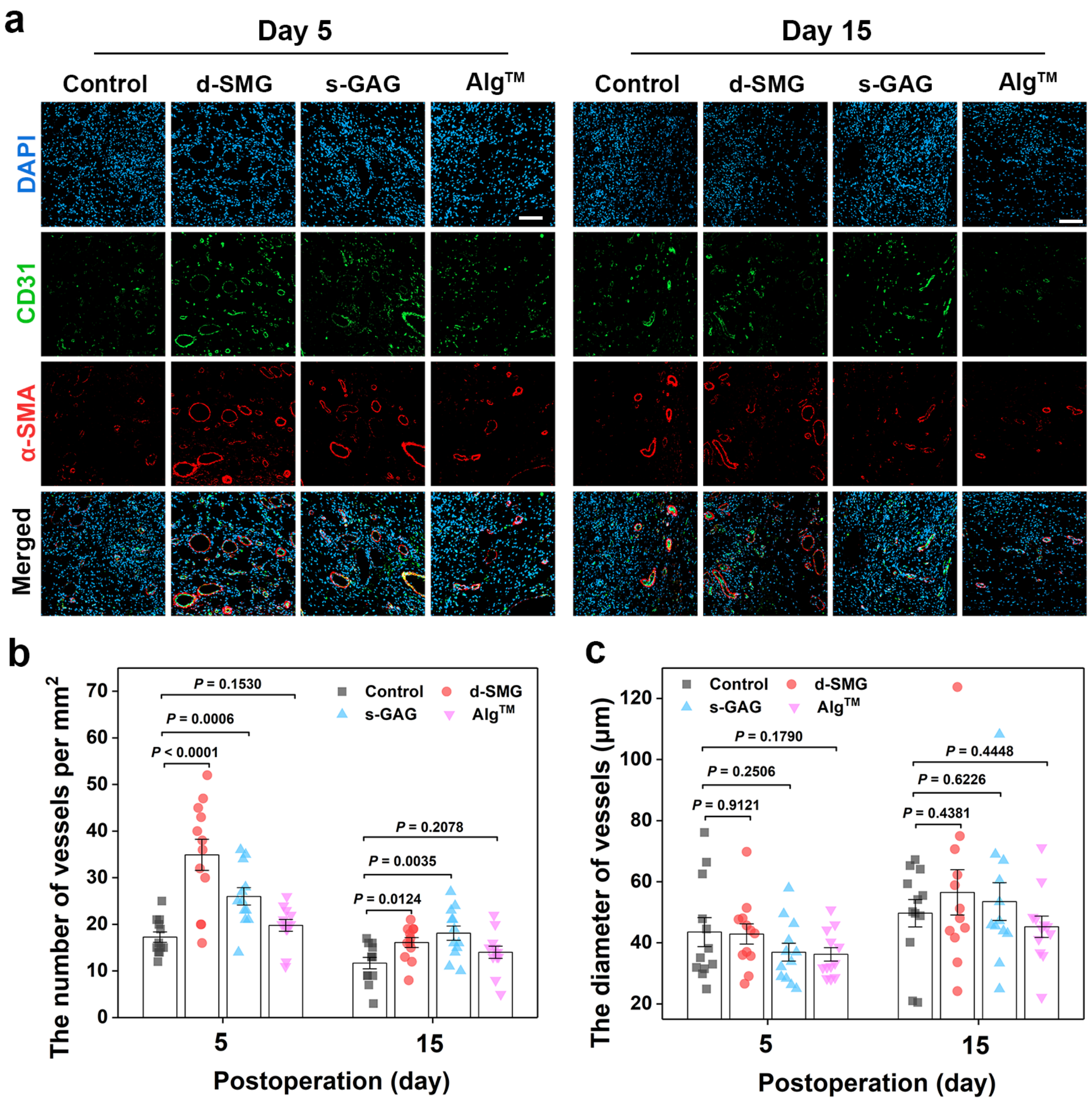

Supplementary Fig. 12. Effects of *A. fulica* d-SMG and s-GAG on angiogenesis in normal rats. a Representative immunostaining images of CD31 and α-SMA in the wound tissue. **b** Quantification of the number of vessels in the wound tissue (*n* = 12 biologically independent samples in each group). **c** Quantification of the diameter of vessels in the wound tissue (*n* = 12 biologically independent samples in each group). For **b** and **c**, two-tailed t test was used. Data are presented as mean values +/- SEM. Scale bar, 100 μm (images in **a**). Source data are provided as a Source data file.

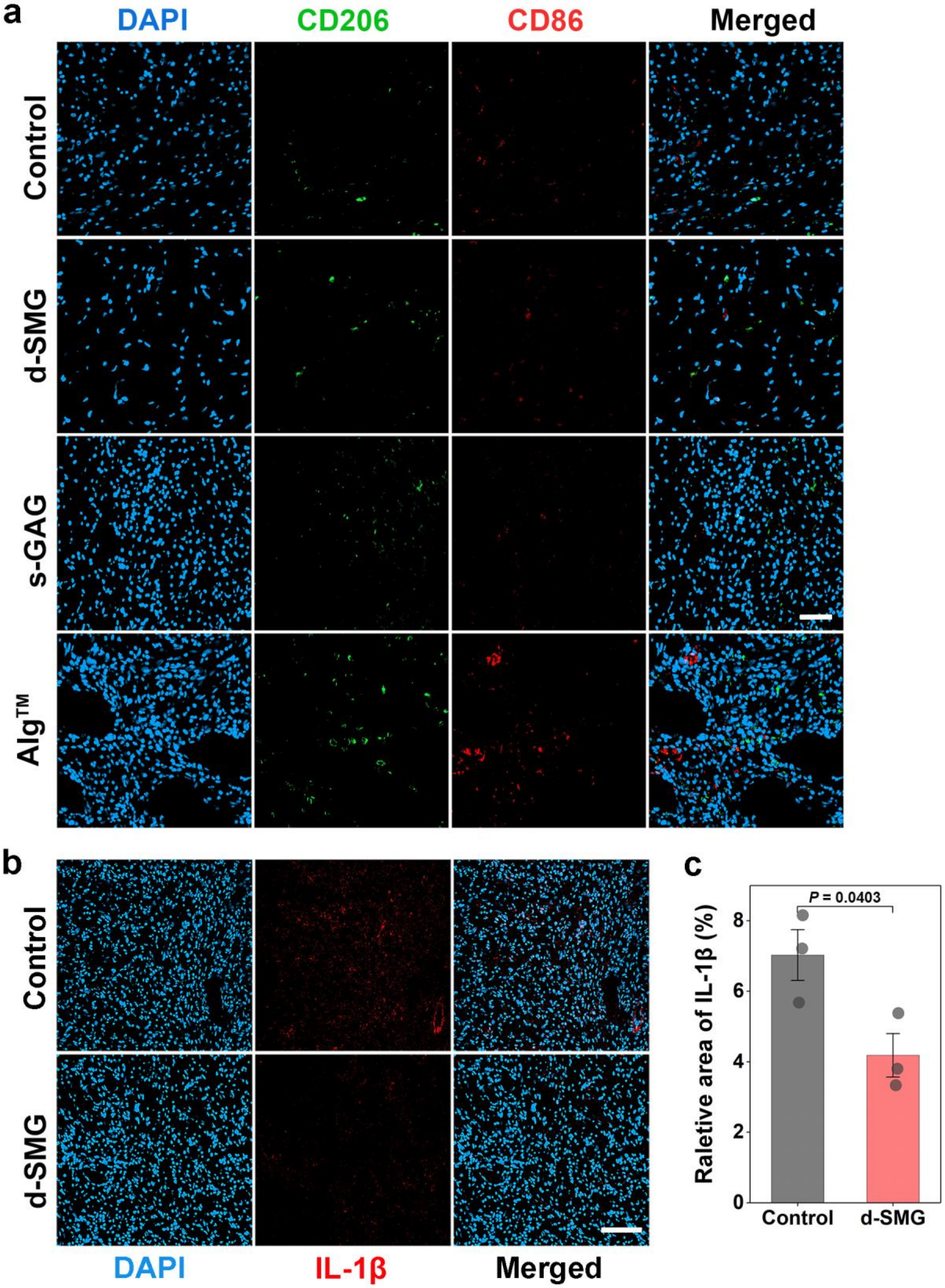
a
DAPI
CD206
CD86
Merged
Control
d-SMG
s-GAG
Alg™
b
Control
d-SMG
DAPI
IL-1β
Merged
c
P = 0.0403
Raletive area of IL-1β (%)
0
2
4
6
8
Control
d-SMG

Supplementary Fig. 13. Effects of *A. fulica* d-SMG on macrophage polarization and inflammation. a Representative immunostaining image of CD206 and CD86 in wound tissue on day 14. **b** Representative immunostaining image of IL-1β in wound tissue on day 7. **c** Quantification of the IL-1β positive area in the wound tissue on day 7 (*n* = 3 biologically independent samples in each group). For **c**, two-tailed t test was used. Data are presented as mean values +/- SEM. Scale bar, 50 μm (images in **a**), 100 μm (images in **b**). Source data are provided as a Source data file.

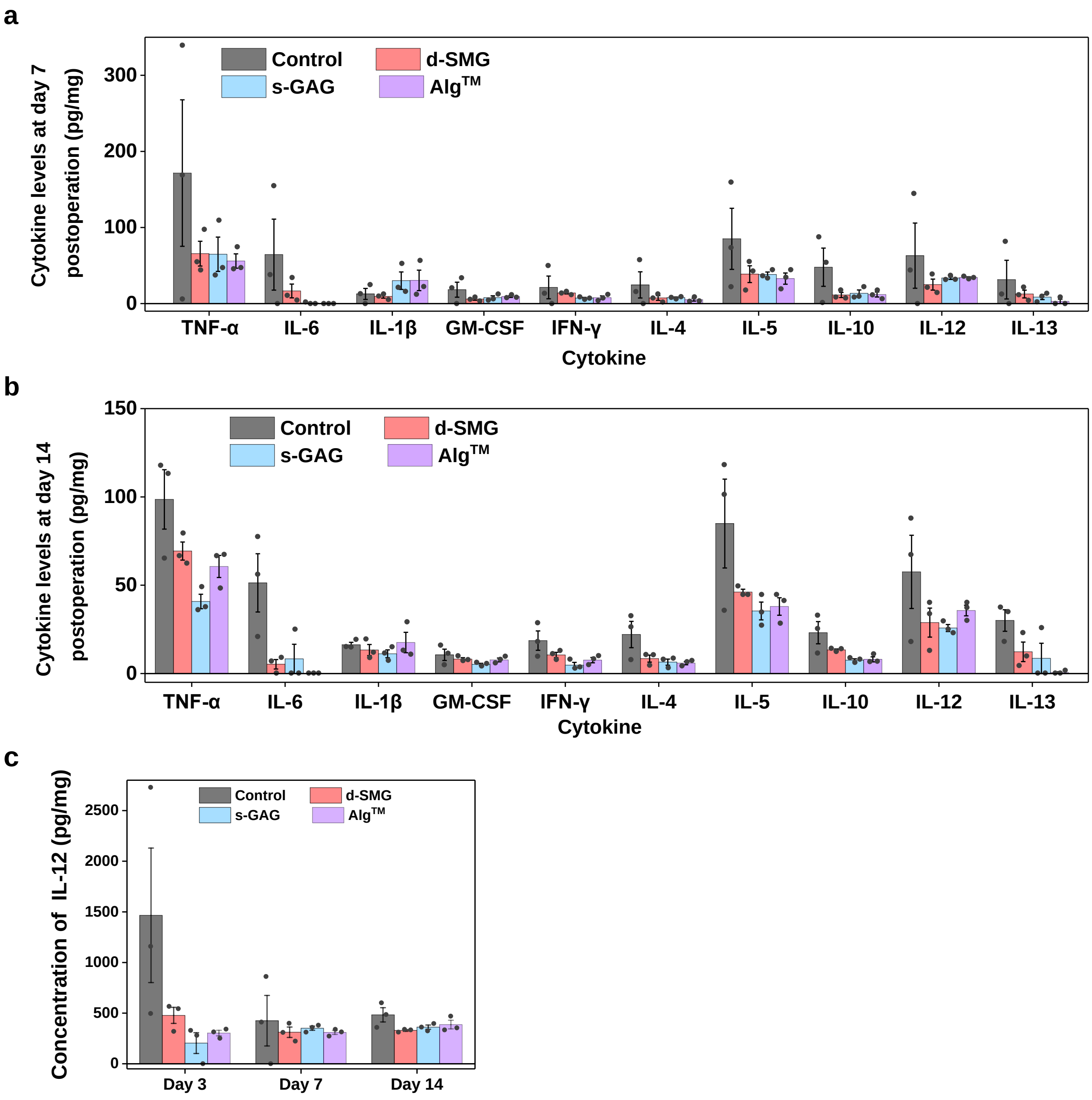

Supplementary Fig. 14. Effects of *A. fulica* d-SMG and s-GAG on cytokine levels of wound tissue. Cytokine levels in diabetic wound tissue on day 7 (**a**) and 14 (**b**) detected by bio-plex assay (*n* = 3 biologically independent samples in each group). **c** Concentration of IL-2 in diabetic wound tissue on day 3, 7, and 14 (*n* = 3 biologically independent samples in each group). For **a-c**, Data are presented as mean values +/- SEM. Source data are provided as a Source data file.

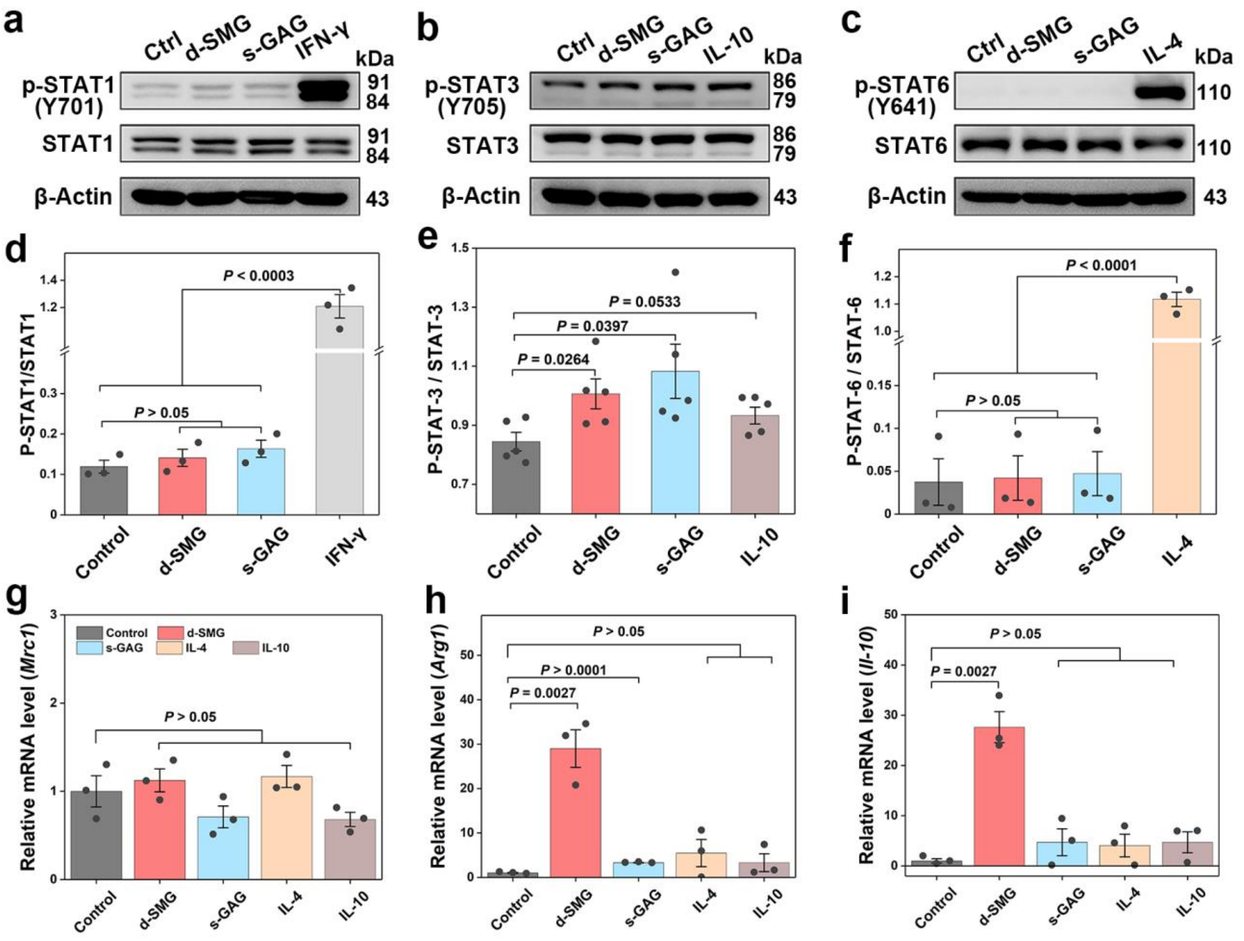

Supplementary Fig. 15. Effects of *A. fulica* d-SMG and s-GAG on inflammatory regulation of macrophages *in vitro*. a–c The protein levels of p-STAT-1 (Y701), p-STAT-3 (Y705), and p-STAT-6 (Y641) in RAW264.7 treated by d-SMG (0.2 mg/mL), s-GAG (0.15 mg/mL), IFN-γ (10 ng/mL), IL-10 (20 ng/mL), and IL-4 (5 ng/mL), assessed by western blot. The phosphorylation or total-protein levels of STAT1, STAT3, and STAT6 were obtained through different gels, because they have the same position on the gel and they cannot be displayed separately. The samples derive from the same experiment and that gels/blots were processed in parallel. **d–f** Relative density of p-STAT-1/STAT-1 (*n* = 3 biologically independent samples in each group), p-STAT-3/STAT-3 (*n* = 5 biologically independent samples in each group), and p-STAT-6/STAT-6 (*n* = 3 biologically independent samples in each group) from western blot images. **g–i** Relative mRNA level in macrophages assessed using real-time PCR (*n* = 3 biologically independent samples in each group). For **d–i**, two-tailed t test was used. Data are presented as mean values +/- SEM. Source data are provided as a Source data file.

a

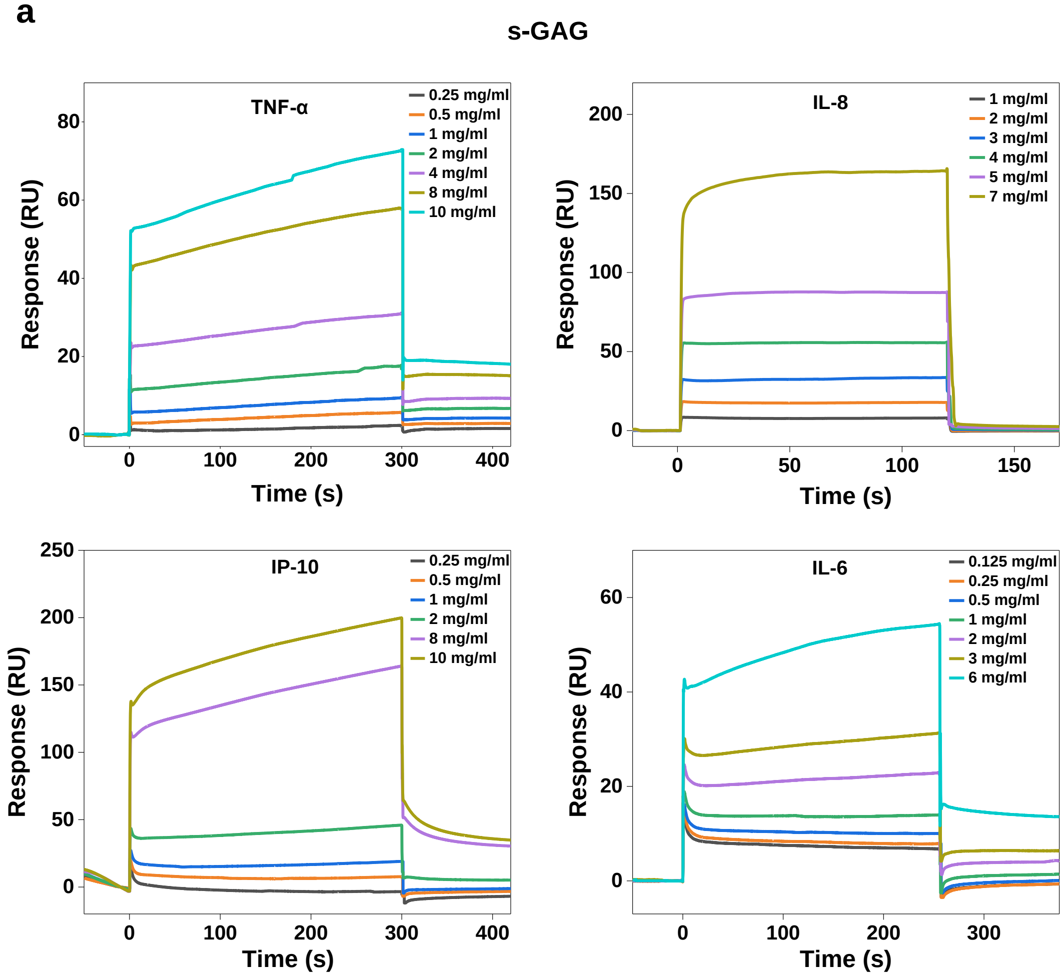
s-GAG
TNF-α
0.25 mg/ml
0.5 mg/ml
1 mg/ml
2 mg/ml
4 mg/ml
8 mg/ml
10 mg/ml
Response (RU)
Time (s)
IL-8
1 mg/ml
2 mg/ml
3 mg/ml
4 mg/ml
5 mg/ml
7 mg/ml
Response (RU)
Time (s)
IP-10
0.25 mg/ml
0.5 mg/ml
1 mg/ml
2 mg/ml
8 mg/ml
10 mg/ml
Response (RU)
Time (s)
IL-6
0.125 mg/ml
0.25 mg/ml
0.5 mg/ml
1 mg/ml
2 mg/ml
3 mg/ml
6 mg/ml
Response (RU)
Time (s)

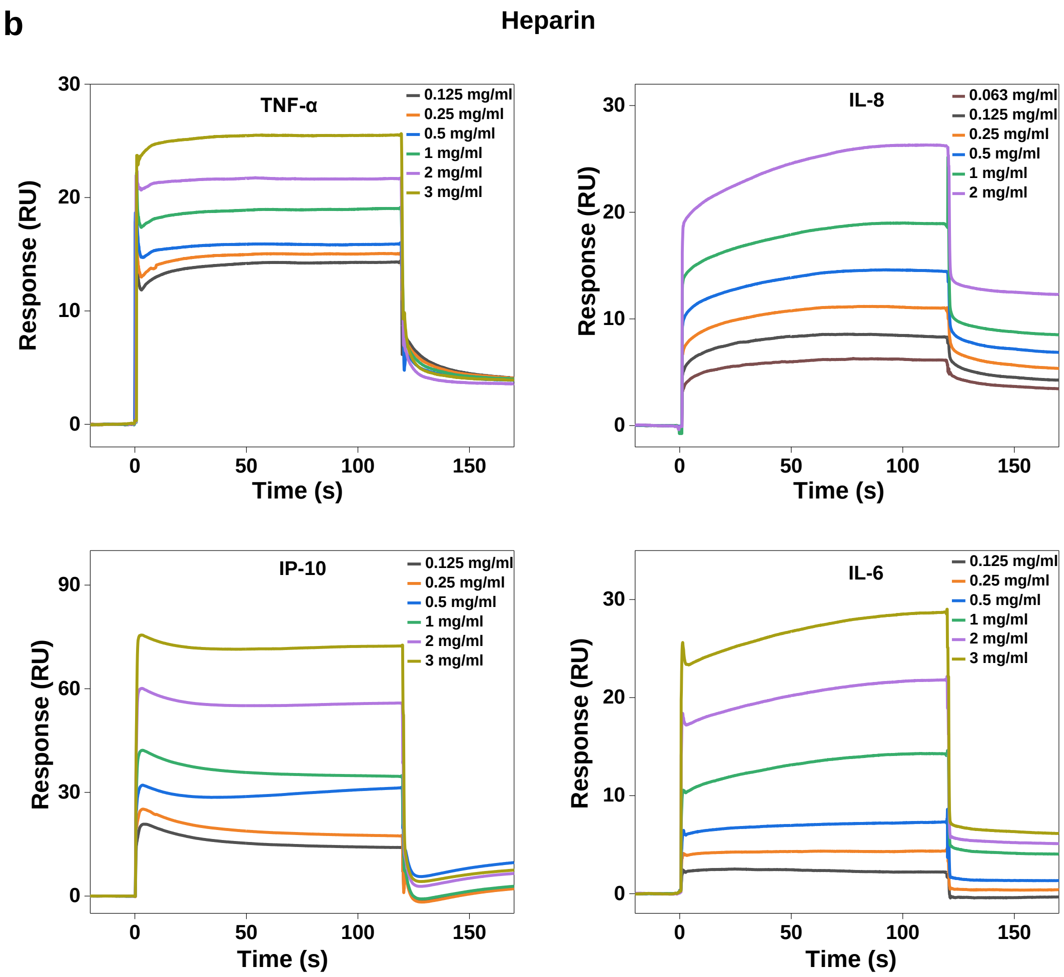

c

The affinity of s-GAG and heparin to cytokines (μM)

GAG	TNF-α	IP-10	IL-8	IL-6
s-GAG	0.336	0.075	0.012	0.171
Heparin	0.233	0.252	0.170	0.609

Supplementary Fig. 16. Binding ability of s-GAG to inflammatory cytokines. The association and dissociation dynamic curves of s-GAG **(a)** and heparin **(b)** binding to immobilized cytokines, detected by SPR technology. **c** The affinity of s-GAG or heparin to cytokines. Source data are provided as a Source data file.

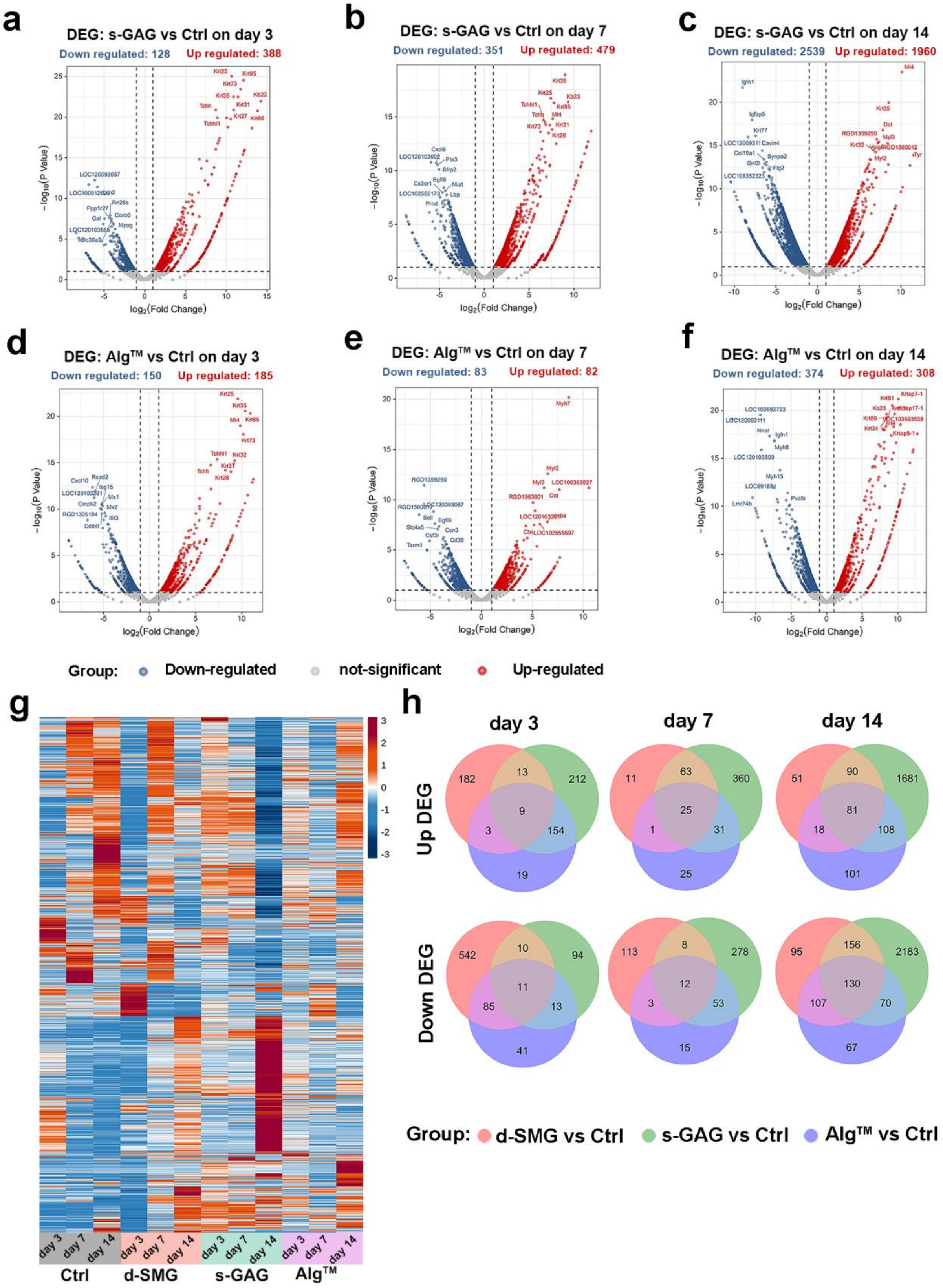
a
DEG: s-GAG vs Ctrl on day 3
Down regulated: 128
Up regulated: 388
b
DEG: s-GAG vs Ctrl on day 7
Down regulated: 351
Up regulated: 479
c
DEG: s-GAG vs Ctrl on day 14
Down regulated: 2539
Up regulated: 1960
d
DEG: Alg™ vs Ctrl on day 3
Down regulated: 150
Up regulated: 185
e
DEG: Alg™ vs Ctrl on day 7
Down regulated: 83
Up regulated: 82
f
DEG: Alg™ vs Ctrl on day 14
Down regulated: 374
Up regulated: 308
−log10(P Value)
log2(Fold Change)
Group: Down-regulated not-significant Up-regulated
g
Ctrl
d-SMG
s-GAG
Alg™
day 3
day 7
day 14
h
day 3
day 7
day 14
Up DEG
182 13 212 9 3 154 19
11 63 360 25 1 31 25
51 90 1681 81 18 108 101
Down DEG
542 10 94 11 85 13 41
113 8 278 12 3 53 15
95 156 2183 130 107 70 67
Group: d-SMG vs Ctrl s-GAG vs Ctrl Alg™ vs Ctrl

Supplementary Fig. 17. Effects of *A. fulica* d-SMG and s-GAG on gene expression in diabetic wound tissue. a–c Volcano plots of the upregulated and downregulated genes in wound tissue after s-GAG treatment for 3 (**a**), 7 (**b**), and 14 (**c**) days. **d–f** Volcano plots of the upregulated and downregulated genes in wound tissue after Alg^{TM} treatment for 3 (**d**), 7 (**e**), and 14 (**f**) days. **g** Heat map of the differentially expressed genes (DEGs) in the wound tissue (FDR < 0.05). **h** Venn diagram of the upregulated and downregulated genes in wound tissue after treatment. For **a-f**, Wald test was used. Source data are provided as a Source data file.

Supplementary Table 1. Structural characteristics of s-GAG from d-SMG

s-GAG	Composition	$-OSO_3^-$ (%)	Molecular weight (kDa)		
			M_w	M_n	PDI (M_w / M_n)
A. fulica d-SMG	IdoA, GlcNAc	15.4	696.7	540.7	1.29
H. lucorum d-SMG	IdoA, GlcNAc, GlcA, Gal	13.6	900.0	725.8	1.24

Supplementary Table 2. Amino acid composition and content of snail mucus and foot (Mean values ± SEM)

Amino acids	*A. fulica* (mg/g)		*H. lucorum* (mg/g)	
	Mucus	Foot	Mucus	Foot
Asp	43.5 ± 1.98	69.2 ± 3.82	58.5 ± 0.40	75.0 ± 8.45
Thr	17.8 ± 0.61	27.8 ± 1.89	27.0 ± 0.10	31.0 ± 3.95
Ser	19.2 ± 0.80	41.3 ± 1.71	26.2 ± 0.30	39.5 ± 4.81
Glu	40.8 ± 1.66	125.1 ± 7.35	57.3 ± 0.67	118.9 ± 13.03
Gly	13.7 ± 0.22	109.4 ± 13.98	25.7 ± 0.20	82.2 ± 14.71
Ala	16.0 ± 0.51	46.0 ± 2.77	22.9 ± 0.07	41.9 ± 4.36
$(Cys)_2$	4.0 ± 0.44	4.8 ± 1.19	7.2 ± 2.48	3.4 ± 1.88
Val	31.6 ±11.03	30.0 ± 1.87	27.0 ± 0.48	31.7 ± 3.80
Met	0.6 ± 0.43	0.3 ± 0.30	1.7 ± 0.25	7.9 ± 0.31
Ile	15.3 ± 0.31	22.3 ±1.58	19.2 ± 0.36	24.0 ± 2.79
Leu	27.5 ± 3.53	49.6 ± 4.25	43.3 ± 3.40	49.4 ± 7.24
Tyr	12.6 ± 0.28	12.9 ± 1.08	19.1 ± 0.39	16.8 ± 1.47
Phe	22.8 ± 1.73	21.9 ± 1.68	30.6 ± 0.18	24.6 ± 3.50
His	16.7 ± 1.52	10.5 ± 1.22	20.7 ± 0.25	11.7 ± 1.62
Lys	18.2 ± 0.69	24.0 ± 9.60	24.2 ± 0.20	37.2 ± 4.79
Arg	22.9 ± 0.90	52.8 ± 11.75	26.3 ± 0.42	56.4 ± 5.96
Pro	17.4 ± 2.79	63.8 ± 6.36	23.4 ± 1.30	45.2 ± 7.71

Supplementary Table 3. Identified proteins (top 95%) in the *A. fulica* d-SMG by LC-MS/MS.

Protein IDs (Uniprot)	Protein	Organism	Unique peptides	Unique sequence coverage	Protein abundance
A0A3G2VHN3	Hemocyanin β	*Cornu aspersum*	4	0.9 %	41.68 %
A0A0B7BT89	Uncharacterized	*Arion vulgari*	8	3.7 %	8.24 %
A0A3G2VHF1	Hemocyanin αN	*Cornu aspersum*	5	1.7 %	6.74 %
A0A3G2VFW4	Hemocyanin αN	*Helix pomatia*	2	0.9 %	6.14 %
A0A3G2VFQ5	Hemocyanin αD	*Cornu aspersum*	2	0.4 %	4.44 %
A0A0B7A203	Uncharacterized	*Arion vulgaris*	1	15.3 %	4.10 %
P35903	Achacin	*Lissachatina fulica*	28	44.8 %	3.65 %
A0A3G2VHT2	Hemocyanin β	*Helix pomatia*	1	0.4 %	3.54 %
A0A0B7A0D2	Uncharacterized	*Arion vulgaris*	2	3.7 %	3.41 %
A0A0B7BCN0	Uncharacterized	*Arion vulgaris*	1	3.3 %	2.29 %
A0A0B7AFX7	Uncharacterized	*Arion vulgaris*	1	2.7 %	2.14 %
A0A0B7APA2	Uncharacterized	*Arion vulgaris*	1	2.9 %	2.06 %
A0A0B6ZQQ7	Haemocyan_bet_s domain-containing	*Arion vulgaris*	1	12.2 %	1.91 %
A0A0B7A255	Uncharacterized	*Arion vulgaris*	2	7.1 %	1.28 %
A0A345S6Z0	Actin, cytoplasmic	*Cepaea nemoralis*	13	38.8 %	1.18 %
A0A0B7AHS6	APH domain-containing	*Arion vulgaris*	1	3.3 %	1.00 %
A0A0B6YGU9	Uncharacterized	*Arion vulgaris*	1	28.2 %	0.98 %
A0A0B7B9A6	Uncharacterized	*Arion vulgaris*	1	3.4 %	0.67 %

Supplementary Table 4. Identified proteins (top 99%) in the *H. lucorum* d-SMG by LC-MS/MS.

Protein IDs (Uniprot)	Protein	Organism	Unique peptides	Unique sequence coverage	Protein abundance
G3FPE6	Hemocyanin αD-subunit	*Helix lucorum*	26	7.9 %	37.14 %
G3FPE5	Hemocyanin β-subunit	*Helix lucorum*	46	18.4 %	28.18 %
G3FPE7	Hemocyanin αN-subunit	*Helix lucorum*	36	18.1 %	23.58 %
A0A3G2VFW4	Hemocyanin αN	*Helix pomatia*	16	5.9 %	3.43 %
A0A3G2VHT2	Hemocyanin β	*Helix pomatia*	18	8.0 %	2.96 %
A0A3G2VHR9	Hemocyanin αD	*Helix pomatia*	13	3.4 %	1.93 %
A0A3G2VHF1	Hemocyanin αN	*Cornu aspersum*	8	3.8 %	0.99 %
A0A3G2VFQ5	Hemocyanin αD	*Cornu aspersum*	13	3.9 %	0.68 %
A0A0B7AMR8	Uncharacterized	*Arion vulgaris*	3	4.4 %	0.64 %
A0A3G2VHN3	Hemocyanin β	*Cornu aspersum*	15	5.6 %	0.16 %
A0A0B7B0J4	Uncharacterized	*Arion vulgaris*	2	4.0 %	0.09 %
A0A345S6Z0	Actin, cytoplasmic	*Cepaea nemoralis*	3	14.6 %	0.06 %
A0A0B6ZIY0	PKS_ER domain-containing	*Arion vulgaris*	2	7.7 %	0.02 %
A0A345S6Y9	Tubulin α chain	*Cepaea nemoralis*	3	10.0 %	0.02 %
A0A0B7C4D3	Tyrosinase_Cu-bd domain-containing	*Arion vulgaris*	1	18.6 %	0.02 %
A0A0B7A345	Phosphoenolpyruvate carboxykinase (GTP)	*Arion vulgaris*	1	2.9 %	0.01 %
A0A0B7B670	Tubulin β chain	*Arion vulgaris*	1	4.0 %	0.01 %
P00994	Isoinhibitor K	*Helix pomatia*	1	24.1 %	0.01 %

Supplementary Table 5. Identified mucins in the *A. fulica* d-SMG by LC-MS/MS.

Protein IDs (Uniprot)	Protein	Organism	Unique peptides	Unique sequence coverage	Protein abundance
A0A2C9JYP9	Mucin-2-like	*Biomphalaria glabrata*	1	3.4%	70.67%
A0A433TRR6	Mucin 96d	*Elysia chlorotica*	1	16.1%	12.40%
A0A2T7PKE9	Mucin-2-like	*Pomacea canaliculata*	4	14.8%	9.15%
A0A2T7PBD6	Mucin-2-like	*Pomacea canaliculata*	1	2.8%	5.69%
A0A2C9L226	Mucin-like protein	*Biomphalaria glabrata*	1	3.7%	1.25%
A0A3S0ZR63	Conserved secreted mucin	*Elysia chlorotica*	2	3.7%	0.85%

Supplementary Table 6. Identified mucins in the *H. lucorum* d-SMG by LC-MS/MS.

Protein IDs (Uniprot)	Protein	Organism	Unique peptides	Unique sequence coverage	Protein abundance
A0A2T7NL50	Mucin-2-like	*Pomacea canaliculata*	1	0.6%	70.12%
A0A0B6ZJM7	Mucin-5AC	*Arion vulgaris*	2	1.3%	7.98%
A0A2T7NM56	Mucin-5AC-like	*Pomacea canaliculata*	1	3%	7.51%
A0A0B6ZSG9	Mucin-5AC-like	*Arion vulgaris*	1	0.9%	3.70%
A0A2C9LWW2	Mucin-1	*Biomphalaria glabrata*	1	3.1%	2.75%
A0A2T7PRF9	Mucin-like protein	*Pomacea canaliculata*	2	1.9%	1.87%
V4AEI7	Mucin 5B	*Lottia gigantea*	1	13.2%	1.74%
A0A2T7PR58	Mucin-like protein	*Pomacea canaliculata*	1	0.7%	1.72%
A0A2C9KEQ3	Mucin-13	*Biomphalaria glabrata*	1	4.2%	1.41%
A0A2T7PHX2	Mucin-5AC-like	*Pomacea canaliculata*	2	2.8%	1.21%

Supplementary Table 7. Identified proteins in the SDS-PAGE of *A. fulica* d-SMG by LC-MS/MS.

SDS-PAGE Fractions	Protein ID (Uniprot)	Protein	Organism	Unique peptides	Unique sequence coverage	Protein abundance
**AF1** (> 130 kDa)	A0A3G2VHN3	Hemocyanin β	*Cornu aspersum*	6	1.9 %	38.31 %
	A0A3G2VHF1	Hemocyanin αN	*Cornu aspersum*	5	1.7 %	9.56 %
	A0A0B7BT89	Uncharacterized	*Arion vulgaris*	5	2.2 %	8.44 %
	A0A3G2VHT2	Hemocyaninβ	*Helix pomatia*	0	0.0 %	6.16 %
	A0A3G2VHR9	Hemocyanin αD	*Helix pomatia*	0	0.0 %	4.95 %
	A0A0B7AMR8	Uncharacterized	*Arion vulgaris*	3	5.4 %	4.53 %
	A0A3G2VFW4	Hemocyanin αN	*Helix pomatia*	4	1.5 %	4.24 %
	A0A0B7APA2	Uncharacterized	*Arion vulgaris*	1	2.9 %	3.06 %
**AF2** (~100 kDa)	G3FPE6	Hemocyanin αD-subunit	*Helix lucorum*	1	0.2 %	33.25 %
	A0A3G2VHF1	Hemocyanin αN	*Cornu aspersum*	7	2.5 %	12.81 %
	A0A3G2VHN3	Hemocyanin β	*Cornu aspersum*	3	0.7 %	10.84 %
	A0A0B7AFX7	Uncharacterized	*Arion vulgaris*	1	2.7 %	8.84 %
	A0A0B7BT89	Uncharacterized	*Arion vulgaris*	5	2.5 %	8.24 %
	A0A3G2VFQ5	Hemocyanin αD	*Cornu aspersum*	2	0.4 %	7.53 %
**AF3** (~60 kDa)	P35903	Achacin	*Lissachatina fulica*	27	43.3 %	62.09 %
	A0A3G2VHN3	Hemocyanin β	*Cornu aspersum*	2	0.3 %	6.42 %
	A0A3G2VFQ5	Hemocyanin αD	*Cornu aspersum*	2	0.4 %	5.72 %
	A0A0B7AFX7	Uncharacterized	*Arion vulgaris*	1	2.7 %	4.00 %
	A0A0B6ZW97	Uncharacterized	*Arion vulgaris*	1	27.4 %	2.84 %
**AF4** (~45 kDa)	A0A0B7AZK1	Transporter	*Arion vulgaris*	1	1.0 %	81.28 %
	A0A345S6Z0	Actin, cytoplasmic	*Cepaea nemoralis*	0	0.0 %	8.56 %
	G3FPE6	Hemocyanin αD-subunit	*Helix lucorum*	0	0.0 %	1.71 %
	A0A3G2VFW4	Hemocyanin αN	*Helix pomatia*	1	0.4 %	1.14 %
	A0A0B7BT89	Uncharacterized	*Arion vulgaris*	2	0.5 %	0.923 %
	A0A220VX78	Actin	*Gibbulinella dewinteri*	1	3.8 %	0.612 %

**AF5** (~15 kDa)	M1HH77	Histone H4	*Chondrina avenacea*	4	30.2 %	25.48 %
	A0A0B7A7U4	Protein kinase domain-containing	*Arion vulgaris*	1	1.5 %	11.80 %
	A0A0B7C5B8	Protein kinase domain-containing	*Arion vulgaris*	1	16.4 %	11.24 %
	A0A0B7AN25	Multiple inositol polyphosphate phosphatase 1	*Arion vulgaris*	1	1.6 %	7.56 %
	A0A0B7A1A5	Golgi apparatus membrane protein TVP23 homolog	*Arion vulgaris*	1	3.2 %	5.34 %
	A0A3G2VFQ5	Hemocyanin αD	*Cornu aspersum*	1	0.3 %	4.87 %
	A0A0B6XU90	XPGN domain-containing	*Arion vulgaris*	1	12.1 %	4.32 %
	A0A0B6Y9Y9	Histone H2B	*Arion vulgaris*	8	45.3 %	3.97 %
	A0A0B6ZNS7	J domain-containing	*Arion vulgaris*	1	2.0 %	3.85 %
	A0A0B6Y975	Histone H2A	*Arion vulgaris*	1	5.5 %	3.19 %

Supplementary Table 8. Identified proteins in the SDS-PAGE of *H. lucorum* d-SMG by LC-MS/MS.

SDS-PAGE Fractions	Protein ID (Uniprot)	Protein	Organism	Unique peptides	Unique sequence coverage	Protein abundance
**HL1** (>130 kDa)	G3FPE6	Hemocyanin αD-subunit	*Helix lucorum*	34	7.8 %	36.54 %
	G3FPE5	Hemocyanin β-subunit	*Helix lucorum*	54	20.2 %	27.00 %
	G3FPE7	Hemocyanin αN-subunit	*Helix lucorum*	40	20.2 %	25.01 %
	A0A3G2VFW4	Hemocyanin αN	*Helix pomatia*	21	7.9 %	3.33 %
	A0A3G2VHR9	Hemocyanin αD	*Helix pomatia*	17	5.0 %	2.63 %
**HL2** (~60 kDa)	G3FPE6	Hemocyanin αD-subunit	*Helix lucorum*	6	1.7 %	18.02 %
	G3FPE7	Hemocyanin αN-subunit	*Helix lucorum*	8	4.7 %	15.04 %
	A0A0B7B016	N domain-containing	*Arion vulgaris*	1	7.0 %	14.28 %
	G3FPE5	Hemocyanin β-subunit	*Helix lucorum*	6	1.9 %	13.51 %
	A0A0B6ZSP8	Uncharacterized	*Arion vulgaris*	1	2.7 %	12.77 %
	A0A0B6YKV0	Uncharacterized	*Arion vulgaris*	1	15.1 %	11.30 %
	A0A0B7A4J6	Katanin p60 ATPase-containing subunit A1	*Arion vulgaris*	1	2.8 %	3.82 %
	A0A3G2VFW4	Hemocyanin αN	*Helix pomatia*	4	1.7 %	1.74 %
**HL3** (~40 kDa)	G3FPE6	Hemocyanin αD-subunit	*Helix lucorum*	7	2.2 %	16.04 %
	A0A0B6ZVB5	Protein kinase domain-containing	*Arion vulgaris*	1	1.5 %	14.64 %
	G3FPE5	Hemocyanin β-subunit	*Helix lucorum*	8	2.9 %	12.60 %
	G3FPE7	Hemocyanin αN-subunit	*Helix lucorum*	6	2.5 %	10.10 %
	A0A0B6Y997	Uncharacterized	*Arion vulgaris*	1	6.1 %	6.56 %
	A0A0B7A4J6	Katanin p60 ATPase-containing subunit A1	*Arion vulgaris*	1	2.8 %	4.91 %
	A0A0B6YX79	Uncharacterized	*Arion vulgaris*	1	17.6 %	4.40 %
	A0A0B6YH37	Uncharacterized	*Arion vulgaris*	1	4.2 %	4.19 %

	A0A0B6ZYW6	Uncharacterized	*Arion vulgaris*	1	23.3 %	3.26 %
	A0A0B6ZDR2	Myosin_tail_1 domain-containing	*Arion vulgaris*	1	2.5 %	3.19 %
	A0A0B7B0A4	AB hydrolase-1 domain-containing	*Arion vulgaris*	1	2.8 %	2.67 %
**HL4** (~30 kDa)	A0A0B7BCQ8	Importin N-terminal domain-containing	*Arion vulgaris*	1	0.9 %	32.55 %
	A0A0B7A456	Uncharacterized	*Arion vulgaris*	1	7.5 %	15.27 %
	A0A0B6ZC31	DUF4200 domain-containing	*Arion vulgaris*	1	1.9 %	11.58 %
	A0A0B6ZKG6	Death domain-containing	*Arion vulgaris*	1	6.2 %	8.15 %
	A0A0B7AH50	Uncharacterized	*Arion vulgaris*	1	19.6 %	6.50 %
	A0A0B7A4J6	Katanin p60 ATPase-containing subunit A1	*Arion vulgaris*	1	2.8 %	5.22 %
	G3FPE6	Hemocyanin αD-subunit	*Helix lucorum*	6	1.5 %	5.11 %
**HL5** (~25 kDa)	A6YM38	Matrilin-like 85 kDa	*Ambigolimax valentianus*	1	1.0 %	39.04 %
	A0A0B7BCQ8	Importin N-terminal domain-containing	*Arion vulgaris*	1	0.9 %	19.15 %
	A0A0B6ZY91	Uncharacterized	*Arion vulgaris*	1	16.0 %	16.63 %
	A0A0B7C6J4	Uncharacterized	*Arion vulgaris*	1	15.6 %	5.93 %
	A0A0B7A456	Uncharacterized	*Arion vulgaris*	1	7.5 %	4.56 %
	A0A0B6Y1H3	Uncharacterized	*Arion vulgaris*	1	6.4 %	3.24 %
	A0A0B7A4J6	Katanin p60 ATPase-containing subunit A1	*Arion vulgaris*	1	2.8 %	1.93 %
**HL6** (~15 kDa)	A0A0B7AQC8	Uncharacterized	*Arion vulgaris*	1	0.9 %	9.93 %
	G3FPE6	Hemocyanin αD-subunit	*Helix lucorum*	8	2.8 %	9.89 %
	A0A0B6YNX5	Uncharacterized	*Arion vulgaris*	1	8.2 %	9.22 %
	G3FPE5	Hemocyanin β-subunit	*Helix lucorum*	4	1.5 %	8.01 %
	G3FPE7	Hemocyanin αN-subunit	*Helix lucorum*	4	1.5 %	6.98 %
	P00994	Isoinhibitor K	*Helix pomatia*	1	24.1 %	5.91 %
	A0A1X9WEF7	Fmrfamide-related peptide 2	*Deroceras reticulatum*	1	4.3 %	5.86 %

	A0A0B6Z4T8	Uncharacterized	*Arion vulgaris*	1	13.5 %	5.78 %
	A0A0B6Z5Q5	SynN domain-containing	*Arion vulgaris*	1	16.7 %	4.77 %
	A0A3G2VHR9	Hemocyanin αD	*Helix pomatia*	2	0.4 %	3.99 %
	A0A0B7AQB0	3-phosphoinositide-dependent protein kinase 1	*Arion vulgaris*	1	1.4 %	3.36 %
	A0A0B7A8X1	Uncharacterized	*Arion vulgaris*	1	3.2 %	3.06 %
	A0A0B6ZFN8	Uncharacterized	*Arion vulgaris*	1	5.1 %	3.02 %
	A0A0B7BL84	Uncharacterized	*Arion vulgaris*	1	2.4 %	2.71 %
	M1HH77	Histone H4	*Chondrina avenacea*	5	50.0 %	2.59 %

Supplementary Table 9. The components and their content in two d-SMGs (Mean values ± SEM)

Component	*A. fulica*	*H. lucorum*
Protein (%)	34.1±1.71	46.0±0.47
s-GAG (%)	15.9±0.11	9.3±0.11
Allantoin (%)	2.6±0.01	1.0±0.02
Na (mg/g)	63.1±1.20	42.4±1.0
Mg (mg/g)	14.7±0.48	6.9±0.18
K (mg/g)	13.1±0.28	8.1±0.10
Ca (mg/g)	26.40±0.38	66.7±1.46
Cu (mg/kg)	364.3±21.10	265.9±40.12
Zn (mg/kg)	42.6±2.90	34.0±5.12
Fe (mg/kg)	66.3±8.68	30.6±4.47
Mn (mg/kg)	21.4±1.22	2.8±0.44
Cl^- (mg/g)	93.2±1.09	66.6±0.86
SO_4^{2-} (mg/g)	14.8±0.65	16.4±0.36
NO_3^- (mg/g)	0.16±0.01	1.1±0.08
Glycolic acid (mg/g)	7.1±0.41	1.0±0.18

Supplementary Table 10 Content of Ca^{2+} and Na^{+} in d-SMG under different treatments.

	Before dialysis		After dialysis in water		After dialysis in 1% EDTA solution	
	A. fulica d-SMG	*H. lucorum* d-SMG	*A. fulica* d-SMG	*H. lucorum* d-SMG	*A. fulica* d-SMG	*H. lucorum* d-SMG
Ca^{2+} (mg/g)	26.4	66.7	10.8	18.6	0.31	0.44
Na^{+} (mg/g)	63.1	42.4	0.41	0.30	0.68	0.69

Supplementary Table 11. Information on the antibodies used in immunofluorescence staining and western blot.

Antibodies	Host	Working Concentration (μg/mL)	Manufacturer	Product code
CD31	Goat	10.0	R&D	AF3628
α-SMA	Mouse	8.0	R&D	MAB1420
CD206	Rabbit	0.418	abcam	ab64693
CD86	Mouse	1.0	abcam	Ab220188
IL-1β	Goat	2.5	R&D	AF-401-SP
STAT-1	Rabbit	1.0	Cell Signaling Technology	#14994
p-STAT-1 (Tyr701)	Mouse	1.0	Santa Cruz Biotechnology, Inc.	sc-136229
STAT-3	Rabbit	1.0	Cell Signaling Technology	#4904
p-STAT-3 (Tyr705)	Rabbit	1.0	Cell Signaling Technology	#9131
STAT-6	Rabbit	1.0	Cell Signaling Technology	#5397
p-STAT-6 (Tyr641)	Rabbit	1.0	Cell Signaling Technology	#56554
β-Actin	Rabbit	1.0	Abclonal	AC026
Anti-Rabbit IgG (AF488)	Donkey	1.0	Jackson Immuno Research	711-545-152
Anti-Goat IgG (AF488)	Donkey	1.0	Jackson Immuno Research	705-545-003
Anti-Mouse IgG (AF594)	Donkey	1.0	Jackson Immuno Research	715-585-150

Supplementary Table 12. mRNA primers used in qPCR

Gene	Organism	Forward sequence	Reverse sequence
β-actin2	Rat/mouse	GGTGTGATGGTGGGTATGGG	ACGGTTGGCCTTAGGGTTCAG
Nos2	Rat	TCCCCTCCTCTACCCTACCA	GCTTCAGTCGGGTGGTTCAT
Tnf	Rat	CCCAGAAAAGCAAGCAACCAG	CTTTCCGGAGGGAGATGTGT
Il1b	Rat	GACAGAACATAAGCCAACAA	ACACAGGACAGGTATAGATTC
Mrc1	Rat	GCACATACCTCAAGAGGCCA	TTCGATGTTGCCCACGAGAT
Arg1	Rat	TTGATGTTGATGGACTGGA	CTCTGGCTTATGATTACCTTC
Jak3	Rat	CAGCATTTGACACCCTGAGC	CTGAAGTCACACCGCCAGAT
Cd163	Rat	AGAAGGAAGCGGACAGAT	CTATGTATCGTGAGCAGACTA
Il10	Rat	AGCTGCAGAGCTGTTTACCA	TCATGGAAGGAGCAACCTGG
Tgfb	Rat	GCAACAACGCAATCTATGA	CAAGGTAACGCCAGGAAT
Vegf	Rat	TTCGAGGAGCACTTTGGGTC	ACTCCCTAATCTTCCGGGCT
Col1a1	Rat	CGAGTATGGAAGCGAAGG	GCAGTGATAGGTGATGTTCT
Col3a1	Rat	CCATTGCTGGAGTTGGAGGT	AGGTCTCTGCAATTCCGAGC
Pdgf	Rat	TAGCACTAGGTTGGGCACTG	TCAGTCGTCAGTGCTAGGGT
Mrc1	Mouse	GTTCACCTGGAGTGATGGTTCTC	AGGACATGCCAGGGTCACCTTT
Arg1	Mouse	CGGGAAGACAATAACTGCACCC	CGGTTAGCAGTATGTTGTCCAGC
Il10	Mouse	CGGGAAGACAATAACTGCACCC	CGGTTAGCAGTATGTTGTCCAGC

Supplementary References

1. Arnow, L. E. Colorimetric determination of the components of 3,4-dihydroxyphenylalaninetyrosine mixtures. *J. Biol. Chem.* **118**, 531-537 (1937).
2. Farndale, R. W., Sayers, C. A., & Barrett, A. J. A direct spectrophotometric microassay for sulfated glycosaminoglycans in cartilage cultures. *Connect. Tissue Res.* **9**, 247-248 (1982).
3. Kosakai, M. Z. & Yosizawa. A partial modification of the carbazole method of Bitter and Muir for quantitation of hexuronic acids. *Anal. Biochem.* **93**, 295-298 (1979).
