# Supplementary material for: A natural biological adhesive from snail mucus for wound repair
Source: Nat Commun. 2023 Jan 24;14:396. doi: 10.1038/s41467-023-35907-4 (PMC9873654; doi:10.1038/s41467-023-35907-4)
Supplement: Supplementary file 3 — Reporting Summary [file 41467_2023_35907_MOESM3_ESM.pdf]

nature portfolio

Corresponding author(s): Mingyi Wu
Last updated by author(s): Jan 4, 2023

Reporting Summary

Nature Portfolio wishes to improve the reproducibility of the work that we publish. This form provides structure for consistency and transparency in reporting. For further information on Nature Portfolio policies, see our Editorial Policies and the Editorial Policy Checklist.

Statistics

For all statistical analyses, confirm that the following items are present in the figure legend, table legend, main text, or Methods section.

n/a	Confirmed	
☐	☒	The exact sample size (*n*) for each experimental group/condition, given as a discrete number and unit of measurement
☐	☒	A statement on whether measurements were taken from distinct samples or whether the same sample was measured repeatedly
☐	☒	The statistical test(s) used AND whether they are one- or two-sided<br>*Only common tests should be described solely by name; describe more complex techniques in the Methods section.*
☒	☐	A description of all covariates tested
☐	☒	A description of any assumptions or corrections, such as tests of normality and adjustment for multiple comparisons
☐	☒	A full description of the statistical parameters including central tendency (e.g. means) or other basic estimates (e.g. regression coefficient) AND variation (e.g. standard deviation) or associated estimates of uncertainty (e.g. confidence intervals)
☐	☒	For null hypothesis testing, the test statistic (e.g. *F*, *t*, *r*) with confidence intervals, effect sizes, degrees of freedom and *P* value noted<br>*Give P values as exact values whenever suitable.*
☒	☐	For Bayesian analysis, information on the choice of priors and Markov chain Monte Carlo settings
☒	☐	For hierarchical and complex designs, identification of the appropriate level for tests and full reporting of outcomes
☒	☐	Estimates of effect sizes (e.g. Cohen's *d*, Pearson's *r*), indicating how they were calculated

Our web collection on statistics for biologists contains articles on many of the points above.

Software and code

Policy information about availability of computer code

Data collection	Storage and loss modulus were determined by an Anton Paar MCR302 rheometer (Germany) with rheocompass software.<br>Immunofluorescence images of wound tissue were acquired using a Carl Zeiss Microscopy GmbH (Axio Scan Z1, Germany).<br>Quantitative Real-Time PCR was performed in a QuantStudio 7 Flex Real-Time PCR detection system (Thermo Fisher, USA).<br>Cytokine concentrations were detected by a flexible Bio-Plex system (#171K1002M, Bio-Rad, USA).<br>For RNA-seq experiments, RNA quality was evaluated by a Nanodrop One spectrophotometer (Thermo Fisher, USA); samples were sequenced by NovaSeq 6000 sequencer (Illumina, USA).<br>The content of allantoin was analyzed by a HPLC system (Agilent 1260, USA).<br>For amino acid composition was analyzed by an amino acid analyzer (A300, Membra Pure GmbH, Germany).<br>Adhesion strength experiments were performed with a universal testing machine (HANDPI Instrument, China).<br>For hemolysis and blood clotting index tests, the absorbance of hemoglobin was determined by a microplate reader (FlexStation 3 Multi-Mode Microplate Reader, Molecular Devices, LLC, USA).<br>Raman spectra were recorded with an inVia Raman microscope (Renishaw, UK) using a 532 nm laser.<br>Immunoreactive proteins were detected using an ECL chemiluminescence system (Clinx Science Instrument, China) with default settings.<br>Surface plasmon resonance was performed with Biacore S200, GE Healthcare.<br>The cell growth status was recorded by IncuCyte S3 Live-Cell Analysis System (Sartorius, Germany).<br>The activated partial thromboplastin time (APTT) was determined using a coagulometer (TECOMC-4000, Germany)<br>qRT-PCR was performed on a QuantStudioTM 7 Flex Real-Time PCR detection system (Thermo Fisher, USA).
Data analysis	Histological staining, immunofluorescence, and wound images were analyzed with ImageJ 1.51j8 (Wayne Rasband, National Institutes of Health, USA).

The kinetic curve of interactions between the s-GAG or heparin and cytokines was fitted by using the Biacore S200 Evaluation Software.
Differentially expressed genes was analyzed using the edgeR software, Heatmaps were generated by the TBtools software (https://github.com/CJ-Chen/TBtools/releases), GO terms and KEGG pathways were identified using KOBAS 2.0.
The MS data of proteins were analyzed and identified based on the protein database of Stylommatophora, the same taxonomic level as Achatina fulica and Helix lucorum (https://www.uniprot.org/, taxonomy_id:6527) using Maxquant (1.6.2.10).
Biacore S200 Control Software was used for affinity test;
SPSS software (version 20.0; IBM, USA) and OriginPro 2017 SR2 (b9.4.2.380) were used for the data analysis and plotting.

For manuscripts utilizing custom algorithms or software that are central to the research but not yet described in published literature, software must be made available to editors and reviewers. We strongly encourage code deposition in a community repository (e.g. GitHub). See the Nature Portfolio guidelines for submitting code & software for further information.

Data

Policy information about availability of data

All manuscripts must include a data availability statement. This statement should provide the following information, where applicable:

- Accession codes, unique identifiers, or web links for publicly available datasets
- A description of any restrictions on data availability
- For clinical datasets or third party data, please ensure that the statement adheres to our policy

All data needed to support the conclusions in the paper are present in the paper and/or the Supplementary Information. Data underlying Figures 1–9 and Supplementary Figures 1–17 is provided with this paper in the Source Data file. The mass spectrometry proteomics data have been deposited to the ProteomeXchange Consortium with the dataset identifier PXD036781 (https://www.ebi.ac.uk/pride/archive/projects/PXD036781). The transcriptome sequencing data used in this study are available in the Gene Expression Omnibus database under accession number GSE206113 (https://www.ncbi.nlm.nih.gov/geo/query/acc.cgi?acc=GSE206113).

Human research participants

Policy information about studies involving human research participants and Sex and Gender in Research.

Reporting on sex and gender	N/A
Population characteristics	N/A
Recruitment	N/A
Ethics oversight	N/A

Note that full information on the approval of the study protocol must also be provided in the manuscript.

Field-specific reporting

Please select the one below that is the best fit for your research. If you are not sure, read the appropriate sections before making your selection.

[x] Life sciences   [ ] Behavioural & social sciences   [ ] Ecological, evolutionary & environmental sciences

For a reference copy of the document with all sections, see nature.com/documents/nr-reporting-summary-flat.pdf

Life sciences study design

All studies must disclose on these points even when the disclosure is negative.

Sample size	No statistical methods were used to predetermine sample size. The sample size was chosen based on previous publications. For instance, the animal sample size can be referred to Ma et al. 2021 (PMID: 34127656), Xiao et al. 2017 (PMID: 28729818). Sample size of other experiments was chosen based on what is common in the field, and what was practical to do. Sample size was determined to be adequate based on the reproducibility between independent experiments.
Data exclusions	No data were excluded from analyses.
Replication	Reproducibility was ensured by sampling from multiple biological replicates, i.e., from multiple rats or from multiple samples. The exact number of replicates in groups or independent biological experiments is mentioned in the figure legends. No results are included that were not observed in multiple experiments. We confirm that all attempts at replication were successful.

Randomization	Male SD rats with similar weight used for normal wound model were randomly allocated into experimental groups. For STZ-induced diabetic rat model, after one week, those with blood sugar lower than 16.7 mM were not included in the experiment, and the qualified diabetic rats were weighed and randomly allocated.
Blinding	Investigators were not blinded to group allocation, because different treatments (such as suture, cyanoacrylate adhesive and hydrogel dressing) in the skin wound can be easily observed even the investigators were blinded to group allocation. Except these special experiments, the investigators were blinded during data collection and/or analysis.

Reporting for specific materials, systems and methods

We require information from authors about some types of materials, experimental systems and methods used in many studies. Here, indicate whether each material, system or method listed is relevant to your study. If you are not sure if a list item applies to your research, read the appropriate section before selecting a response.

Materials & experimental systems

n/a	Involved in the study	
☐	☒	Antibodies
☐	☒	Eukaryotic cell lines
☒	☐	Palaeontology and archaeology
☐	☒	Animals and other organisms
☒	☐	Clinical data
☒	☐	Dual use research of concern

Methods

n/a	Involved in the study	
☒	☐	ChIP-seq
☒	☐	Flow cytometry
☒	☐	MRI-based neuroimaging

Antibodies

Antibodies used

Antibodies/Host/Working Concentration (μg/mL)/Manufacturer/Product code
1. CD31/Goat/10.0/R&D/AF3628
2. Alpha-Smooth Muscle Actin (α-SMA)/Mouse/8.0/R&D/MAB1420
3. CD206/Rabbit 0.418/abcam/ab64693
4. CD86/Mouse/1.0/abcam Ab220188
5. IL-1β/Goat/2.5/R&D/AF-401-SP
6. STAT-1/ Rabbit/ 1.0/ CST/ #14994
7. Phospho-STAT-1 (Tyr701)/ Mouse/ 1.0/ Santa Cruz Biotechnology, Inc. /sc-136229
8. STAT-3/ Rabbit /1.0 /CST /#4904
9. Phospho-STAT-3 (Tyr705) /Rabbit/ 1.0 /CST/ #9131
10. STAT-6/ Rabbit/ 1.0 /CST /#5397
11. Phospho-STAT-6 (Tyr641)/ Rabbit /1.0/ CST/ #56554
12. β-Actin /Rabbit /1.0/ Abclonal /AC026
13. Anti-Rabbit IgG (AF488)/Donkey/1.0/Jackson Immuno Research/711-545-152
14. Anti-Goat IgG (AF488)/Donkey/1.0 /Jackson Immuno Research/705-545-003
15. Anti-Mouse IgG (AF594)/Donkey/1.0/Jackson Immuno Research/715-585-150

Validation	Antibodies validated by manufacturer were used for immunohistochemistry:<br>1. Goat anti-CD31: https://www.rndsystems.com/cn/products/mouse-rat-cd31-pecam-1-antibody_af3628<br>2. Mouse anti-Alpha-Smooth Muscle Actin: https://www.rndsystems.com/cn/search?keywords=%CE%B1-SMA<br>3. Rabbit anti-CD206: https://www.abcam.cn/mannose-receptor-antibody-ab64693.html<br>4. Mouse anti-CD86: https://www.abcam.cn/cd86-antibody-c861146-ab220188.html<br>5. Goat anti-IL-1β: https://www.rndsystems.com/cn/products/mouse-il-1beta-il-1f2-antibody_af-401-na<br>6. Rabbit anti-Stat1: https://www.cellsignal.cn/products/primary-antibodies/stat1-d1k9y-rabbit-mab/14994?site-search-type=Products&N=4294956287&Ntt=%2314994&fromPage=plp&_requestid=7468177<br>7. Mouse anti-Phospho-Stat1 (Tyr701) : https://www.scbt.com/p/p-stat1-antibody-py701-4a?requestFrom=search<br>8. Rabbit anti-Stat3: https://www.cellsignal.cn/products/primary-antibodies/stat3-79d7-rabbit-mab/4904?site-search-type=Products&N=4294956287&Ntt=%234904&fromPage=plp&_requestid=7468710<br>9. Rabbit anti-Phospho-Stat3 (Tyr705) : https://www.cellsignal.cn/products/primary-antibodies/phospho-stat3-tyr705-antibody/9131?site-search-type=Products&N=4294956287&Ntt=%239131&fromPage=plp&_requestid=7468906<br>10. Rabbit anti-Stat6: https://www.cellsignal.cn/products/primary-antibodies/stat6-d3h4-rabbit-mab/5397?site-search-type=Products&N=4294956287&Ntt=%235397&fromPage=plp&_requestid=7469051<br>11. Rabbit anti-Phospho-Stat6 (Tyr641): https://www.cellsignal.cn/products/primary-antibodies/phospho-stat6-tyr641-d8s9y-rabbit-mab/56554?site-search-type=Products&N=4294956287&Ntt=%2356554&fromPage=plp&_requestid=7469180<br>12. Rabbit anti-β-Actin: https://abclonal.com.cn/catalog/AC026<br>13. Anti-Rabbit IgG (AF488): https://www.jacksonimmuno.com/catalog/products/711-545-152<br>14. Anti-Goat IgG (AF488): https://www.jacksonimmuno.com/catalog/products/705-545-003<br>15. Anti-Mouse IgG (AF594): https://www.jacksonimmuno.com/catalog/products/715-585-150

Eukaryotic cell lines

Policy information about cell lines and Sex and Gender in Research

Cell line source(s)	L929 (GDC0034) cell line was purchased from China Center for Type Culture Collection, Chinese Academy of Sciences. RAW264.7 (SCSP-5036) cell line was purchased from National Collection of Authenticated Cell Cultures, Chinese Academy of Sciences.
Authentication	Cell lines were authenticated by providers. All cell lines showed normal size, karyotype, morphology, and no contamination was observed.
Mycoplasma contamination	All cell lines were tested negative for mycoplasma contamination using MycAway Plus-Color One-Step Mycoplasma Detection Kit (Yeasen Biotechnology Co. Ltd., Shanghai, China).
Commonly misidentified lines<br>(See ICLAC register)	No commonly misidentified cell lines were used.

Animals and other research organisms

Policy information about studies involving animals; ARRIVE guidelines recommended for reporting animal research, and Sex and Gender in Research

Laboratory animals	Adult male Sprague Dawley rats (8–12 weeks) were purchased from Hunan Shrek Jingda Experimental Animal Co., Ltd. (Hunan, China). Achatina fulica snails (20–23 g) were from the Fangyuan Snail Farm (Xiangyang, China). Helix lucorum snails (15–19 g) were from Lver Agricultural Science and Technology Park (Luoyang, China).
Wild animals	This study did not involve wild animals.
Reporting on sex	All Sprague Dawley rats in this study were male. Sex difference was not considered in this study as referred to published literatures.
Field-collected samples	This study did not involve field-collected samples.
Ethics oversight	All animal experiments were approved by the Research Ethics Committee of the Kunming Institute of Botany (SYXK-K2018-0005), Chinese Academy of Sciences.

Note that full information on the approval of the study protocol must also be provided in the manuscript.
